# Intracellular Spatial Transcriptomic Analysis Toolkit (InSTAnT)

**DOI:** 10.21203/rs.3.rs-2481749/v1

**Published:** 2023-01-27

**Authors:** Anurendra Kumar, Alex W. Schrader, Ali Ebrahimpour Boroojeny, Marisa Asadian, Juyeon Lee, You Jin Song, Sihai Dave Zhao, Hee-Sun Han, Saurabh Sinha

**Affiliations:** 1 College of Computing, Georgia Institute of Technology, Atlanta, GA, 30332, USA; 2 The Wallace H. Coulter Department of Biomedical Engineering, Georgia Institute of Technology, Atlanta, GA, 30332, USA; 3 H. Milton Stewart School of Industrial & Systems Engineering, Georgia Institute of Technology, Atlanta, GA, 30318, USA; 4 Department of Chemistry, University of Illinois Urbana-Champaign, Urbana, IL, 61801, USA; 5 Department of Statistics, University of Illinois Urbana-Champaign, Urbana, IL, 61820, USA; 6 Department of Computer Science, University of Illinois Urbana-Champaign, Urbana, IL, 61801, USA; 7 Department of Cell and Developmental Biology, University of Illinois Urbana-Champaign, Urbana, IL, 61801, USA; 8 Carl R. Woese Institute for Genomic Biology, University of Illinois Urbana-Champaign, Urbana, IL, 61801, USA

## Abstract

Imaging-based spatial transcriptomics technologies such as MERFISH offer snapshots of cellular processes in unprecedented detail, but new analytic tools are needed to realize their full potential. We present InSTAnT, a computational toolkit for extracting molecular relationships from spatial transcriptomics data at the intra-cellular resolution. InSTAnT detects gene pairs and modules with interesting patterns of mutual co-localization within and across cells, using specialized statistical tests and graph mining. We showcase the toolkit on datasets profiling a human cancer cell line and hypothalamic preoptic region of mouse brain. We performed rigorous statistical assessment of discovered co-localization patterns, found supporting evidence from databases and RNA interactions, and identified subcellular domains associated with RNA-colocalization. We identified several novel cell type-specific gene co-localizations in the brain. Intra-cellular spatial patterns discovered by InSTAnT mirror diverse molecular relationships, including RNA interactions and shared sub-cellular localization or function, providing a rich compendium of testable hypotheses regarding molecular functions.

## Introduction

A grand challenge in biology is to understand how molecules and cells cooperatively perform higher-level processes and how these processes are coordinated to perform life functions. An emerging approach to this question involves using single-cell sequencing technologies which allows profiling of cellular composition and states at unprecedented resolution^1,2^. Spatial omics technologies further bolster this approach by characterizing the spatial organization of molecules and cells, providing insights into their functional organization. Various analytic tools have been developed to extract biological insights from spatial data, such as detecting spatially variable genes^3,4^, identifying spatial domains and their cellular compositions^5–7^, reconstructing spatial gradients in developing organs^8^, or inferring cell-cell interactions^9,10^. Most of these efforts, however, have focused on cell-level or coarser resolution analyses. For grid-based spatial encoding technologies, such as Visium^11^ or DBiT-seq^12^, the resolution is limited by grid size, which is often larger than cells. Even with single-molecule resolution technologies^13–17^, tissue-scale analyses mostly set the unit of analysis to be a cell^6,7^. The focus on cell-level analyses is likely due to the straightforward interpretations they provide, such as cellular arrangements around diseased phenotypes^18^, cellular interactions^9^, and spatial context-dependent cell functions^19^.

Analyzing subcellular patterns of transcriptome expression can add new dimensions to our understanding of cell functions. RNA localization underlies important cellular processes such as transcriptional regulation^20–22^, translational regulation^23^, and protein localization^24,25^. The few studies that perform subcellular analyses on spatial transcriptomics data show exciting potential. For instance, Xia et al^26^ estimated RNA velocity based on the relative distribution of genes in nuclei versus cytoplasm^27,28^ while Bento^29^, a recently proposed analytical toolkit, identifies subcellular domains where a gene tends to appear and was used to explore molecular interactions involving RNA Binding Proteins (RBP)^30,31^.

Despite this initial progress, the subcellular spatial landscape of RNA molecules remains largely unexplored, especially for single-molecule resolution maps, which tap into a new dimension of spatial architecture: spatial organization of molecules in a cell. A facet of the subcellular spatial landscape that naturally merits attention is RNA-RNA proximity. Molecular interactions are mediated by physical contacts; thus, the distance profile of molecular pairs can be used to infer potentially interacting pairs. More broadly, RNA-RNA proximity may arise due to various reasons: direct interactions between molecules, interactions with common mediator molecules, and interactions with a subcellular structure, etc. Each of these sources of proximity in turn implicates biological functions and molecular mechanisms. Even though single-molecule resolution spatial transcriptomics data offer an unprecedented window into this world of sub-cellular organization, there are no analysis tools to probe these phenomena in a large-scale and unbiased fashion.

Here, we introduce Intra-cellular Spatial Transcriptomics Analysis Toolkit (InSTAnT), a set of methods for extracting subcellular localization patterns of RNA. It identifies gene pairs whose transcripts tend to appear within distance *d* significantly more than by chance (“*d*-colocalized pairs”) and reports the cellular domains where they appear. Additional modules characterize the *d*-colocalized pairs by their cell-type specificity and tissue-scale spatial modulation, and also identify colocalizing gene modules. InSTAnT employs formal statistical procedures to account for various sources of confounding such as overall transcript abundance, which is critical for highlighting gene pairs whose transcript-proximity has biological implications. Demonstrative applications of the InSTAnT toolkit to MERFISH data on a human osteosarcoma cell line and on mouse hypothalamic preoptic region identified hundreds of *d*-colocalized gene pairs with low estimated false positive rates and high reproducibility between replicates and data sources. The identified gene pairs exhibit biologically relevant higher order characteristics such as specificity to cell types or non-random spatial distribution in the tissue sample. We also found evidence of their possible relationship to RNA-RNA or RNA-protein interactions, pathway-level co-functionality, and localization to domains such as nuclear speckles. Our results suggest that InSTAnT can recover known biology and generate new hypotheses about the functional role of RNA spatial localization. We believe that the statistical concept of *d*-colocalization introduced in this work will serve as a fundamental unit of subcellular spatial transcriptomics analyses, similar to how co-expression analysis has served as a core concept of transcriptomics analysis.

## RESULTS

### Overview of InSTAnT

**Figure 1.**
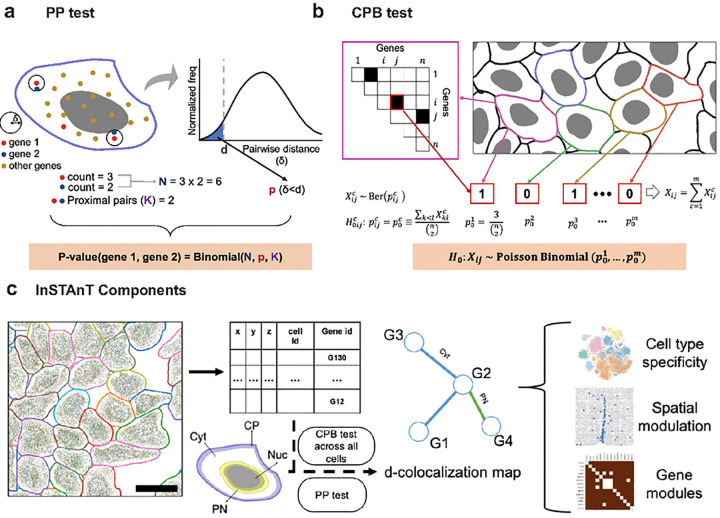
Schematic of InSTAnT. **(a)** Schematic of Proximal Pair (PP) test to detect if transcripts of a gene pair (gene 1, gene 2) tend to occur near each other in a single cell, *d* denotes the distance threshold used. A histogram of distances (*δ*) between transcript pairs (regardless of gene identity) in the cell is used to calculate the background probability of a transcript pair being located within distance *d* of each other *p(δ < d)*, and the number of such proximal pairs (K) of the pair (gene 1, gene 2) is assessed using a Binomial test, **(b)** Simplified schematic of Conditional Poisson Binomial (CPB) test. For each cell, gene pairs found to be significant by the PP test are noted (triangular matrix, top left). For a gene pair *i*, *j*, the random variable $X_{ij}^{c}$ indicates if it is significant under the PP test and is assumed to have a Bernoulli distribution with parameter $p_{0}^{c}$. estimated as the fraction of all pairs that are significant in that cell. The sum *X*_*ij*_ of $X_{ij}^{c}$ over all cells follows a Poisson Binomial distribution. The CPB test further adjusts $p_{0}^{c}$ to be dependent on the genes *i*, *j* (not illustrated here), **(c)** Schematic showing functionalities of the InSTAnT toolkit. The input is spatial transcriptomics data in the form of a text file with spatial coordinates and gene identifier of each transcript, with transcripts in the same cell being tagged with a common cell identifier. At the core of the toolkit is the PP test, applied to each cell separately, and the CPB test, applied on the collection of cells, resulting in a *d*-colocalization map whose nodes are genes and edges are significantly *d*-colocalized gene pairs; edges are also annotated with the cellular region where the represented gene pair tends to colocalize. Perinuclear (PN) region is defined as including 2.5 microns on either side of the nuclear membrane, while Cell Periphery (CP) is defined as regions within 4 microns of the cell membrane. Remaining regions are designated as Cytosol (Cyt) or Nucleus (Nuc). The global map can then be further analyzed to identify gene pairs whose *d*-colocalization is specific to a cell type, is spatially modulated at the tissue level, or to identify modules of genes that colocalize with each other.

InSTAnT is a suite of statistical tools for spatial transcriptomics analysis at sub-cellular resolution. It can discover intracellular spatial patterns involving transcripts of multiple genes, leading to hypotheses regarding their functional relationships. At its heart is a statistical test to detect “proximal pairs” of genes by analyzing the spatial coordinates of transcripts of a set of genes within that cell, available from single-molecule resolution spatial transcriptomics technologies^13–17^. Specifically, the “Proximal Pairs” (PP) test determines if transcripts of a gene pair, in a given cell, are located within a distance threshold *d* significantly more often than expected by chance (Figure 1a). The null expectation may vary from cell to cell, depending on cell size and RNA density, so it is calculated empirically based on the distances between all detected pairs of transcripts in a cell regardless of gene identities. The test provides a p-value for each gene pair, representing its departure from this expectation (Methods). The scale parameter *d* is user-configurable, allowing the user to probe the spatial texture at different scales. The PP test can be implemented in either two- or three-dimensions (PP-3D), depending on whether or not data are available from multiple z-planes (Methods).

We define a *“d*-colocalized” gene pair to be a pair that is detected as proximal pair by the PP test in significantly many cells. This gives us increased confidence in a spatial relationship between the two genes. Like other statistical phenomena such as differential expression of a gene or co-expression of a gene pair, d-colocalization may serve as a starting point for discovery of underlying biological relationships. To detect *d*-colocalization, InSTAnT provides a test called “Conditional Poisson Binomial” (CPB) test that assigns a p-value to a gene pair based on the number of cells in which it is found to be a proximal pair. This test is based on a Poisson Binomial distribution and allows for the fact that different cells have varying numbers of proximal pairs due to varying transcript counts and spatial distributions (Figure 1b, Methods). Initially, we noticed certain genes to feature among the reported *d*-colocalized pairs far more frequently, due to their high expression (Supplementary Figure 1). The CPB test de-emphasizes pairs involving such genes by adjusting the null distribution of each pair to account for the global *d*-colocalization frequency of the involved genes (Methods).

Through the PP and CPB tests, InSTAnT unbiasedly identifies gene pairs with a tendency for spatial proximity, at the level of individual cells (proximal pairs) and at the level of all cells (*d*-colocalized pairs), respectively. The InSTAnT suite is available as a python package with routines that return PP test results for every cell and CPB test results across all cells, for each gene pair. To assist with biological interpretation of the detected spatial relationships, it can annotate each *d*-colocalized gene pair with the cellular regions where its proximal transcripts tend to be found: nuclear, peri-nuclear, cytosolic and peri-membrane. InSTAnT reports the primary and secondary regions that has most PP counts for each gene pair across all cells (Figure 1c, Methods). InSTAnT also implements additional analyses to study *d*-colocalization in intact tissue, where a number of complex biological factors such as heterogeneity of cell types and interactions among neighboring cells are at play. These factors may influence, or be influenced by, RNA-RNA proximity patterns. InSTAnT can assess the cell-type specificity of *d*-colocalized gene pairs, characterize tissue-level spatial modulation of *d*-colocalization patterns, and identify modules of genes that are all frequently colocalized across multiple cells (Figure 1c).

### InSTAnT finds gene-gene relationships with high accuracy

**Figure 2.**
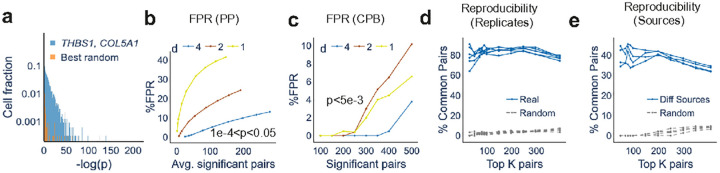
Assessment of InSTAnT on U2OS MERFISH data. **(a)** Histogram of −log(p-value) obtained from PP Test for the gene pair *THBS1*, *COL5A1* over 3237 cells. Also shown is the histogram of −log(p-value) for the best gene pair after randomizing gene identities on the results of PP test for each cell. The best gene pair corresponds to the pair having highest number of cells with proximal pair, **(b)** Estimates of false positive rates (FPR) at varying p-value thresholds for PP test. For each distance threshold d, we vary the p-value threshold and compare the average number of significant pairs per cell on randomized data to the average number on real data. The estimated FPR (y) is plotted against the average number of significant pairs detected per cell, **(c)** Estimates of FPR at varying number of detected pair obtained by varying p-value thresholds for CPB test. The number of significant pairs on randomized data is compared to the number (at the same p-value threshold) on real data to obtain an FPR estimate at that threshold, which is plotted against the number of significant pairs, **(d)** Reproducibility of CPB test results across replicates of a dataset. For each pair of replicates (out of four), the K most significant pairs (by CPB test) in either replicate are compared, and the percentage of shared pairs (out of K) reported (blue). The exercise was repeated for randomized versions of the replicates to obtain random baselines (grey), **(e)** Reproducibility of CPB test results across different datasets. Each replicate of the Moffit et al.^32^ MERFISH data set was compared to our MERFISH data for U2OS to obtain percentages of common *d*-colocalized pairs (blue). Corresponding random baselines are shown in grey.

We first applied InSTAnT to the published MERFISH data on human osteosarcoma cells (U2-OS), which profiles 130 genes in 3237 cells with an average of 1243 transcripts per cell^32^ (Methods). Through the analysis, we identified ‘proximal pairs’ within each cell and ‘*d*-colocalized pairs’ across all cells with high accuracy. We calculated false positive rates (FPRs) by applying InSTAnT to a random baseline dataset established by permuting the gene labels of all transcripts within each cell, which recapitulates the spatial patterns of the original data but not the gene-gene relationships. As shown in Figure 2b (blue), the PP test identifies hundreds of significant proximal pairs with an estimated FPR below 10%. Smaller values of the scale parameter *d* yielded larger FPR values (red and lemon, Figure 2B), suggesting lower sensitivity of the test and/or lesser frequency of proximal pairs in this regime. We found similar operating characteristics for the CPB test (Figure 2c). Throughout our paper, we use FPR to select p-value threshold for PP (FPR<10%) and CPB Test (FPR<1%). We arrived at similar estimates of accuracy through an entirely different approach that exploits presence of “blank” gene probes in the data (Methods and Supplementary Figure 2). Overall, our tests suggested that hundreds of gene pairs exhibit the *d*-colocalization phenomenon, out of all ~8,500 pairs possible with 130 genes.

The CPB test had sufficient power to identify 404 *d*-colocalized gene pairs at an FPR of < 1% (p < 0.001), with *d* = 4 μm (~5% of the diameter of an average cell) (**Supplementary Table 1**). An example of a highly significant pair thus found is *THBS1-COL5A1*, with a p-value below ~1E-300, the smallest number reportable by the program. This pair appeared as a proximal pair (PP test p-value < 0.01) in ~74% of the 3,147 cells where both genes were detected. Figure 2a shows the distribution of PP test p-values for this gene pair in all cells, compared to the distribution of the strongest p-value in each cell after shuffling gene labels. The comparison illustrates how the CPB test detects the persistent appearance of a proximal pair across many cells.

Our next assessment focused on the replicability of *d*-colocalization findings across four biological replicates of the U2OS data set available from Moffit et al.^32^. We identified the most significant gene pairs (CPB test, *d* = 4 μm) in each replicate and observed that ~80% of the top 50 – 400 gene pairs are common between replicates (Figure 2d), supporting the reproducibility of the reported pairs. The same assessment performed after randomizing each of the four replicate data sets yielded a baseline level of ~5% or less for the replicability expected by chance.

We also tested the extent to which *d*-colocalization phenomena persist across independent MERFISH experiments. For this, we generated the spatial transcriptome map of U2OS cells using our home built MERFISH platform (Methods). We used InSTAnT to identify *d*-colocalized gene pairs from our dataset and compared the top K (for varying values of K) gene pairs between the Moffitt et al. and our data. As shown in Figure 2e, about 30–40% of the identified gene pairs are shared between these two studies, across the range of K examined. The same analysis with randomized versions of the two datasets reveals < 5% of the gene pairs to be shared between studies. As another reference point, a similar comparison of the top co-expressed gene pairs (detected using correlation of cellular transcript counts) shows similar or lesser extent of commonality between the two studies (Supplementary Figure 3). Taken together, these reproducibility analyses suggest that the *d*-colocalized gene pairs reported by InSTAnT capture real biological phenomena or relationships.

### InSTAnT constructs global *d*-colocalization maps

**Figure 3.**
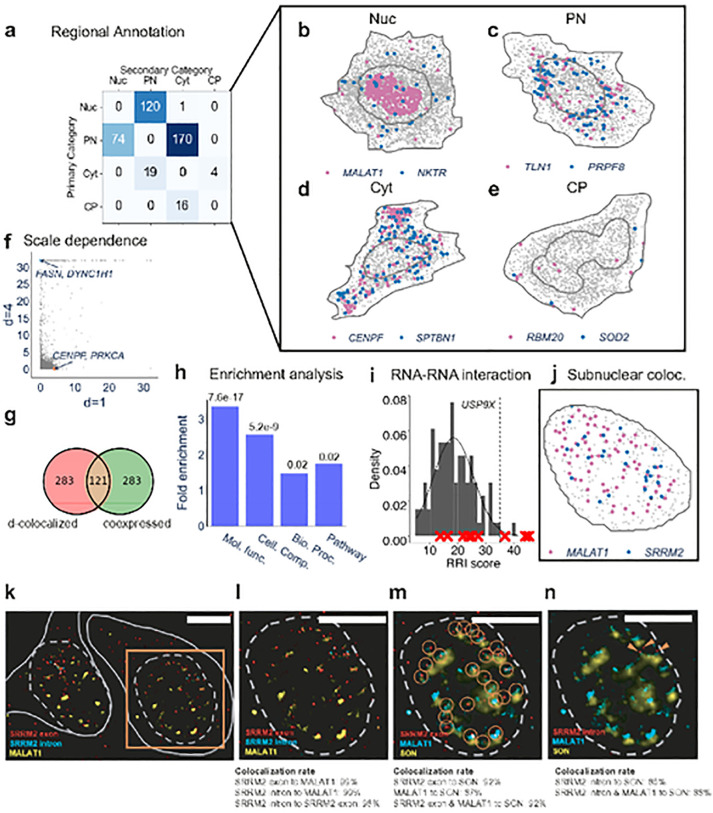
InSTAnT results on U2OS data. **(a)** Regional annotation (nuclear, perinuclear, cytoplasm or perimembrane) of all *d*-colocalized gene pairs. Proximal pairs of a *d*-colocalized gene pair across all cells are recorded and aggregated over all cells to obtain the most and second-most frequent regional annotations, **(b-e)** Examples of *d*-colocalized gene pairs annotated as nuclear (b). perinuclear (c). cytosolic (d) and cell periphery (e) respectively. Shown is one of many cells in which the respective gene pair was significant by the PP test. **(f)** Negative log p-value from the CPB test for all gene pairs, at *d*=1 micron and *d*=4 micron. An example of a gene pair specific to each d is highlighted, **(g)** Overlap of the set of *d*-colocalized gene pairs with co-expressed gene pairs. Co-expressed gene pairs are identified based on Pearson correlation of whole-cell transcript counts, and the top 404 pairs are taken to match the size of the *d*-colocalization map. **(h)** Hypergeometric test is performed to show enrichment of set of *d*-colocalized pairs with set of functionally related gene pairs. A gene pair is functionally related if both genes are annotated with same GO terms or Kegg pathway, **(i)** The grey histogram shows the null distribution of RRI scores for *USP9X*. calculated using the RNAs of genes with which it is *not d*-colocalized. This provides a null distribution of RRI scores, which is then used to test if the genes with which it *is d*-colocalized (defined here as those with 10 smallest p-values under the CPB test) are enriched for high (greater than 35) RRI scores. The test yields a p-value of le-4. due to the 3 genes with high RRI scores being included among the 10 *d*-colocalized partners of *USP9X*. **(j)** Nucleus of a cell showing transcripts of *MALAT1* and *SRRM2*. The PP test p-value for this nucleus is 4.3e-19. **(k)** *SRRM2* exon (red). *SRRM2* intron (cyan), and *MALAT1* (yellow). RNAs labeled with smFISH probes in fixed U-2 OS cells. Dashed gray lines indicate the nuclear boundaries, and solid gray lines indicate cytosolic boundaries. **(l)** Selected nuclear region shown in the orange box in (k). showing high co-localization rate of *SRRM2* exon mRNAs with *MALAT1* IncRNAs in the nucleus. As expected, the *SRRM2* intron puncta co-localize with *SRRM2* exon puncta. **(m)** *SRRM2* exon mRNA (red). *MALAT1*1ncRNA (cyan), and SON protein (yellow) labeled in the same nucleus as (I). Orange circles indicate co-localization of *SRRM2* exon puncta and *MALAT 1* puncta. most of the *SRRM2* exons co-locatize with *MALAT1. SON* protein was selected to label nuclear speckles. Many co-localized RNA pairs are nearby to SON protein, **(n)** Similar as (m). plotting *SRRM2* intron (red) with *MALAT1* IncRNA and SON protein, orange arrows indicate *SRRM2* intron puncta that co-localize with *MALAT1* puncta. Similar to *SRRM2* exon puncta. *SRRM2* intron puncta tend to be near SON protein. Co-localization rate calculated using 13 cells. Scale bars for (k-n) is 10 μm.

The 404 *d*-colocalized gene pairs found using CPB test at d = 4 μm (**Supplementary Table 1**) constitute the global *d*-colocalization map. InSTAnT provides annotations of the cellular regions where each gene pair tends to colocalize, revealing perinuclear and nuclear colocalization as most frequent (Figure 3a–c, Supplementary Figure 4). We also noted many gene pairs to colocalize in the cytosolic (23) or cell periphery (16) regions (see Figures 3d,e for examples), though far less often than the other two categories.

A *d*-colocalization map is expected to capture different biology at different values of *d*. The maps created from the published U2OS data at *d* = 1 μm (**Supplementary Table 2**) and 4 μm revealed substantial complementarity (Figure 3f): while 152 pairs were common to the top 404 significant pairs of either map, 197 of the pairs in the *d* = 4 map had CPB test p-value > 0.1 in the *d* = 1 map, and 167 gene pairs were similarly exclusive to the *d* = 1 map. Two examples of such scale-specific pairs are *FASN-DYNC1H1* (only with *d* = 4) and *CENPF-PRKCA* (only with *d* = 1). (See Supplementary Figure 5 for a more detailed report of their scale-dependence.) These results illustrate scale-dependence of the colocalization phenomenon and suggest that multiple types of biological relationships may underlie its detection.

The d-colocalization map probes a new type of information and may represent yet-to-be-explored phenomena. Reconstructing gene-gene co-expression networks is a common analysis performed with non-spatial single cell RNA-seq data^33^. To test if the global *d*-colocalization map reflects such co-expression networks or if it reveals a different type of relationship, we derived a co-expression network from cell-level transcript counts in the same MERFISH data and found it to share ~ 30% of gene pairs with the colocalization map (Hypergeometric test p-value 6.3e-70) (Figure 3g, **Supplementary Table 3**). Over 70% of the pairs in either “co-expressed” or “colocalized” set were exclusive to that set, suggesting that *d*-colocalization relationships are not revealed through conventional co-expression analysis.

In addition to constructing a basic global map, InSTAnT can run the PP test in a “intra-nucleus” mode where the analysis, including null distribution estimation, is limited to subnuclear transcripts. This mode is critical for detecting subnuclear phenomenon. The default (whole-cell) mode assumes the null distribution as uniform throughout a cell, disregarding the selective enrichment of certain genes in subcellular regions. Thus, nucleus-enriched genes, such as long noncoding RNAs (lncRNAs), often dominate detected co-localized pairs. For example, 89 of the 404 pairs in the U2-OS global co-localization map involved the lncRNA *MALAT1*, which is the most nucleus-enriched gene (89% in nucleus). The intra-nucleus mode effectively removes such bias. As expected, many gene pairs detected by the whole-cell mode have far stronger p-values than the intra-nucleus mode due to the greater number of transcripts examined. (Supplementary Figure 6). However, we also observed a significant number of gene pairs that were assigned greater statistical significance in the intra-nucleus analysis. Such pairs promise to reveal biologically meaningful spatial patterns within nuclei, as might arise for instance from colocalization of a gene pair to subnuclear structures, organelles and domains.

### *d*-colocalization maps suggest functional relationships in U2OS cells

One plausible mechanism for *d*-colocalization is direct or indirect interaction between two RNAs. To test this, we computed an RNA interaction score (“RRI score”) for all gene pairs using RNAplex^34^. To capture the greater proximity expected of interacting RNAs, we set *d* to 200 nm (MERFISH resolution between pixels is 167nm). For each gene, we tested if its transcript tends to have a higher RRI score for the RNAs of its *d*-colocalization partners (Methods) and found this to be the case for eight genes out of 130 (FDR <= 0.2) (**Supplementary Table 4**). An example is shown in Figure 3i, focusing on *USP9X*. In summary, this analysis suggests that RNA-RNA interactions may underlie some of the relationships in a global *d*-colocalization map at a suitably small value of the scale parameter.

Furthermore, we found that the d-colocalized gene pairs were enriched with functionally related gene pairs, where we define a gene pair to be functionally related if both genes are present in the same KEGG pathway or are annotated with same biological process, molecular function, or cellular component GO terms (Methods) (Figure 3h). The highest enrichment happened with molecular function GO terms, where 461 functionally related pairs and 403 d-colocalized pairs had an overlap of 67 pairs. Interestingly, all 67 pairs in this intersection were annotated with the term “protein binding”. Overall, these results suggest that *d*-colocalization of a gene pair may have biological consequences such as colocalization of their protein products or protein binding to form a ribonucleoprotein (RNP) complex.

The intra-nuclear analysis shows that a d-colocalization map can detect RNA-protein interactions as well as identify subnuclear domains. The most prominent pair in the intra-nucleus analysis at d=2 μm is *MALAT1-SRRM2*, with a CPB test p-value of 2.50e-16 (see Figure 3j), while the corresponding p-value in the whole-cell analysis is 0.51 (see marked point in Supplementary Figure 1c). It is detected as a proximal pair in 11% of the nuclei, the most for any pair involving either *SRRM2* or *MALAT1*. Notably, the SRRM2 protein is a key marker of nuclear speckles (NS), organizing NS formation via liquid condensation^35^, and the lncRNA *MALAT1* is well known to be localized to NS^36^, suggesting that the detected intra-nuclear *d*-colocalization of these two RNAs may be related to their colocalization in NS. This is an intriguing possibility though, since NS localization of SRRM2 protein does not imply or necessitate a similar localization of its mRNA. To see whether lncRNA *MALAT1* and mRNA *SRRM2* colocalize near NS, we co-stained *MALAT1*, *SRRM2* mRNA, and SON in U2-OS cells using single molecule FISH and immunostaining (Figure 3k–n). For *SRRM2*, the probes were designed separately for intron and exon to distinguish pre-mRNA and mRNA. As expected, all the *SRRM2* intron signals directly overlap with the *SRRM2* exon signals. Consistent with the InSTAnT result, most *SRRM2* RNAs are d-colocalized with *MALAT1* in *SRRM2* positive cells (99±1%, N=13 cells). The overlaid SON signals show that most d-colocalized *MALAT1-SRRM2* pairs are within 1 μm distance from NS (92±4% for *SRRM2* exon and 88±8% for *SRRM2* intron). It is well known that SRRM2 protein signals overlaps with SON signals^35^; thus, our result shows the d-colocalization of *SRRM2* mRNA and pre-mRNA with SRRM2 proteins in nucleus. Further, these results suggest that d-colocalization maps can be used to infer subcellular domains, such as NS.

### InSTAnT analysis of brain MERFISH data reveals cell type-specific spatial patterns

**Figure 4.**
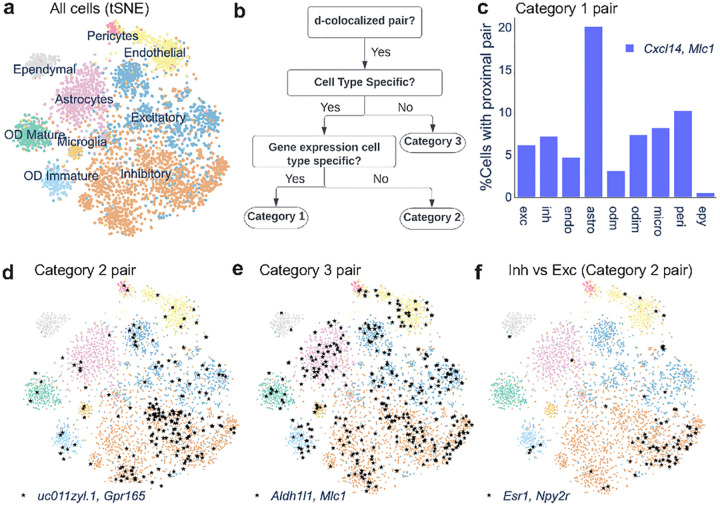
Cell type specificity of *d*-colocalized pairs in mouse hypothalamus preoptic region. **(a)** t-SNE plot of all cells annotated with cell type assignments obtained from M off it et al^37^. The gene count for each cell is aggregated by summing their transcript count across seven z-slices. **b)** Flow chart showing how a *d*-colocalized pair is classified into one of three categories depending on whether it is a proximal pair (PP test) in cells specifically of a cell type and whether either gene is a marker of that cell type, **(c)** Example of a category 1 pair, found to be a proximal pair in many cells of different types but significantly more frequently in astrocytes. Shown is the percentage of cells of each type where the gene pair is significant in the PP test. The gene pair is of category 1 because both genes are marker genes, **(d)** Example of a category 2 pair, specific to inhibitory neurons. Each black star is a cell where the pair was significant under PP test, **(e)** Example of a category 3 pair, which is a proximal pair in cells of many types and not specific to any type, **(f)** Example of a gene pair specific to inhibitory neurons compared to excitatory neurons. (Cell type specificity was defined based on a two-way comparison here, in contrast to the one-versus-all comparison used for examples in c-e.)

We next used InSTAnT to analyze MERFISH data^37^ on 5149 cells from the hypothalamic preoptic region in mouse. This brain dataset includes nine different cell types (Figure 4a), so InSTAnT’s *d*-colocalization maps can be used to give additional insight into cell type differences. The data feature seven z-planes and were thus analyzed with the PP-3D test of proximal pairs. We set the scale parameter *d* to 2 μm, corresponding to ~5% of average cell diameter. The analysis identified 474 gene pairs with CPB test p-value < 1e-5 (**Supplementary Table 5**) (estimated FPR < 1%). This map was further processed with downstream InSTAnT modules for cell type specificity and spatial modulation.

InSTAnT uses a sequence of statistical tests (Methods, Figure 4b) to place *d*-colocalized gene pairs into one of three categories of cell type-specificity. Category 3 comprises pairs that were not associated with any cell type (Bonferroni corrected hypergeometric p-value >= 0.05, **Supplementary Table 5**). Pairs that did appear as proximal pairs more frequently in some cell types than expected were further divided into two classes – those where cell type specificity may arise simply because one of the genes in the pair is expressed specifically in that cell type (Category 1) and those whose association goes beyond what would be expected from the cell-type specificity of either gene’s expression (Category 2) (Methods). We identified 5 gene pairs in Category 2, specific to inhibitory neurons, excitatory neurons, and endothelial cells (**Supplementary Table 6**), while 203 pairs fell in Category 1.

Gene pairs with strong *d*-colocalization signal in each category captured interesting biological processes involving their counterpart protein-protein interactions. In Category 1, the genes *Aqp4* (Aquaporin 4), *Cxcl14* (CXC motif chemokine ligand 14) and *Mlc1* (Modulator of VRAC current 1) show strong pairwise *d*-colocalization associated with astrocytes (CPB test p-value < 1.9E-149, Hypergeometric p-value of cell type association< 2.23E-12). As illustrated for the pair *Cxcl14-Mlc1* in Figure 4c, these pairs are frequently colocalized in cells of most types, but with a higher frequency in astrocytes, leading to the statistically detected specificity. *Cxcl14* transcripts are known to be enriched in and possibly locally translated in peripheral astrocyte processes (PAPs)^38^. We speculate that *Mlc1* transcripts are also subject to local translation in PAPs, leading to the *d*-colocalization of *Cxcl14* and *Mlc1*. Additionally, MLC1 protein forms a complex with AQP4 in cultured astrocytes^39^ and localizes to the cell membrane^38,40^ providing the functional implication of *Mlc1-Aqp4* RNA *d*-colocalization.

In Category 2 (Figure 4d), transmembrane proteins *Gpr165* (G protein-coupled receptor 165) and *uc011zyl.1* (adhesion molecule with Ig like domain 2) form the *d*-colocalized pair most significantly associated with inhibitory neurons, while *Gpr165* and *Omp* (Olfactory marker protein, known to be involved in olfactory signaling processes^41^ form a *d*-colocalized pair specific to excitatory neurons (Supplementary Figure 7). This example illustrates that different *d*-colocalized pairs involving a common gene (Gpr165) can statistically mark different cell types. We observed 61 *d*-colocalized pairs in Category 3. *Aldh1l1-Mlc1* is the strongest pair (CPB test p-value: 1.6E-169), detected as a proximal pair in 7% of all cells, but these cells are not enriched for any one cell type (Figure 4e). This example suggests that *d*-colocalization can capture biological relationships that transcend any cell type-specific function of the constituent genes.

We also identified *d*-colocalized gene pairs that marked cellular function, in particular inhibitory versus excitatory neurons (**Supplementary Table 6**). For instance, *Esr1* (estrogen receptor 1) and *Npy2r* (Neuropeptide Y receptor Y2) are *d*-colocalized specifically in inhibitory neurons compared to excitatory neurons (p-value 5.9E-8, see Figure 4f). Prior work shows that the expression of these two genes underlies a social behavioral switch in virgin mice via activation of a specific subtype of neurons^42^, suggesting the functional implication of *Esr1-Npy2r d*-colocalization.

### InSTAnT reveals tissue-level spatial modulation of *d*-colocalization patterns

Brain tissue is well-known to be spatially heterogeneous, so we applied InSTAnT’s spatial modulation analyses to study how *d*-colocalization varies across the mouse hypothalamic preoptic region. Such tissue-level spatial modulation has been reported for individual gene expression^3,4^. In contrast, here we used InSTAnT to identify spatial patterns of transcript colocalization.

**Figure 5.**
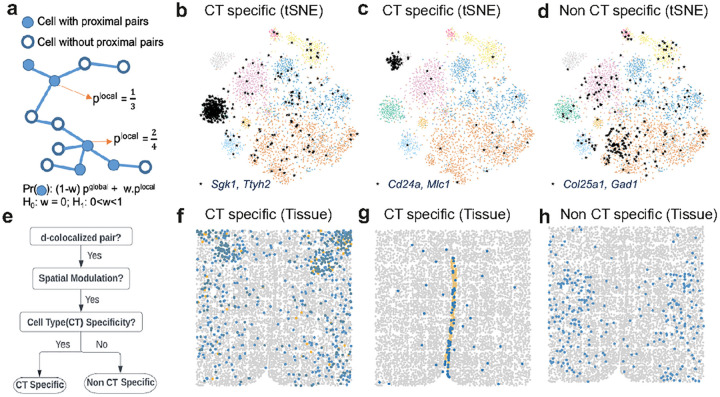
Spatial modulation of *d*-colocalized pairs in mouse hypothalamus preoptic region. **(a)** Probabilistic graphical model to detect spatially modulated gene pair. In a graph where nodes represent cells and edges represent spatial proximity, each cell is first flagged based on whether the gene pair is significant by PP test in that cell. The likelihood function is a product over all cells of a weighted sum *of p*^local^, the local density of flagged cells in cell’s neighborhood, and *p*^*global*^, free parameter. The weight *w* is also a free parameter. A likelihood ratio score is computed to compare this model to a null model where the local (spatial) information is not used, **(b)** t-SNE plot of a spatially modulated *d*-colocalized gene pair *Sgk1*-*Ttyh2* showing that it is a proximal pair (black stars) significantly more often in Mature Oligodendrocytes (OD) and is significant in other cell types. (See Figure 4a for cell type annotations.) **(f)** Cells in spatial coordinates, shown in blue if the gene pair of (b) *Sgk1*-*Ttyh2* is a proximal pair, in orange if the cell is Mature OD but Sgk*1*-*Ttyh2* is not a proximal pair, and in grey otherwise. **(c,g)** t-SNE plot (c) and spatial plot (g) showing a spatially modulated *d*-colocalized gene pair *Cd24a*-*Mlc1* significant specifically in cells of one cell type (ependymal cells) and not significant in other cell types. **(d,h)** t-SNE plot (d) and spatial plot (h) of a gene pair *Col25a1*-*Gad1* that is a proximal pair across several cell types, **(e)** Flowchart showing how a spatially modulated *d*-colocalized pair is categorized based on its cell type specificity.

The analysis is based on a probabilistic model for calculating data likelihood under the hypothesis of spatial modulated *d*-colocalization, for a specific gene pair. The probabilistic model (Figure 5a) examines whether the PP test detects significant colocalization in a cell and assumes that the probability of this happening depends on observed colocalization in neighboring cells, rewarding spatially clustered distributions of cells that support colocalization. Such a model is then contrasted with a null model lacking spatial dependence, resulting in a log likelihood ratio (LLR) score being assigned to each gene pair in the *d*-colocalization map. Pairs above a threshold (obtained using randomization of data) are then designated as spatially modulated. This yielded 99 spatially modulated pairs out of the 474 pairs in the global map (**Supplementary Table 7**). A similar analysis for U2OS data yielded 11 gene pairs out of 404 *d*-colocalized pairs in the corresponding global map. The stark difference in extent of spatially modulation detected is expected, since intercellular communication plays a greater role in the biology underlying the brain data compared to cell line data.

Forty nine of the 99 spatially modulated pairs in the brain data exhibited *d*-colocalization in a cell type-specific manner (p-value 5E-6, Bonferroni corrected p-value < 0.05). For instance, the gene pair *Sgk1*-*Ttyh2* – the strongest spatially modulated pair (LLR 305, Figure 5f) – colocalizes far more frequently in mature oligodendrocytes than others (Hypergeometric test p-value 1.5e-248, Figure 5b). Sgk1 is a serine/threonine-protein kinase that mediates oligodendrocyte plasticity in mouse in response to stress^43,44^ and regulates several ion channels^45^, while Ttyh2 is a chloride channel noted for its transcriptional response to chronic stress in mouse oligodendrocytes^46^. It is plausible that the oligodendrocyte-specific *d*-colocalization results from a co-functional relationship between these two genes. The pair *Slc17a6*-*Syt4* is the second strongest spatially modulated *d*-colocalized pair (LLR 191), detected in six different cell types but highly specific to excitatory neurons (Supplementary Figure 8). In contrast to these two examples where *d*-colocalization is significant in multiple cell types but more frequent in one cell type, the pair *Cd24a-Mlc1* exhibits spatially modulated *d*-colocalization (LLR 79, Figure 5g) that is significant only in ependymal cells (Figure 5c).

We also found 15 spatially modulated gene pairs whose *d*-colocalization is not specific to any cell type (Hypergeometric test p-value > 0.05 for every cell type), the strongest being *Col25a1-Gad1* (LLR 97, Figures 5d,h). Col25a1 is generated by different types of neurons, i.e., inhibitory as well as excitatory, and interneurons in retino-recipient regions of the mouse brain, in a Gad1-dependent pattern^47^. In summary, the above examples of spatially modulated *d*-colocalization provide a rich pool of potential functional relationships for future exploration.

### InSTAnT reveals modules of genes colocalizing with each other

We asked if the significant gene pairs found by InSTAnT point to the existence of *d*-colocalization “modules”, i.e., sets of genes whose transcripts tend to occur in subcellular proximity, across many cells, drawing inspiration from co-expression module discovery^48^. Colocalized gene modules, if found, may reflect ribonucleoprotein complex formation^29,49^ or other shared functional relationships^30^.

**Fig 6.**
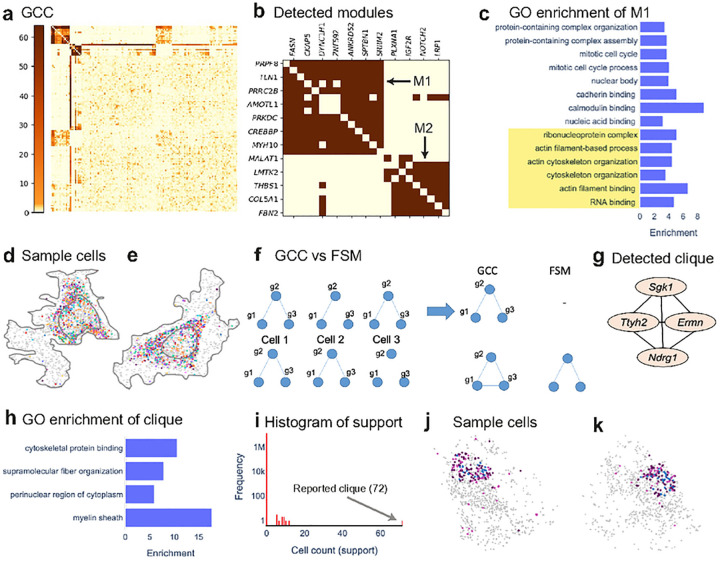
Gene Module Discovery. **(a) Global Colocalization Clustering** (GCC): Global *d*-colocalization map for U2OS data, represented as a matrix of −log(p-value) of CPB test for gene pairs, is subjected to hierarchical clustering to reveal two gene modules, **(b)** Closer view of the two modules (M1, M2) discovered by GCC, shown after thresholding p-values at 1e-3 (FPR<1%). **(c)** Gene Ontology (GO) terms enriched in gene module M1, shown with the fold enrichment over random expectation. (Criterion for selection: Fisher exact p-value < 0.03) **(d, e)** Two cells illustrating spatial distribution of transcripts of M1 genes (colored dots) along with all other transcripts (grey). Each color corresponds to a gene, **(f)** Schematic illustration of difference between Global Colocalization Clustering (GCC) and Frequent Subgraph Mining (FSM). In each row, the three graphs on the left show proximal pair relationships (edges) involving genes g1, g2, g3, in three different cells. In either case, GCC reports the 3-gene module as the global map includes each of the three gene pairs. FSM, on the other hand, finds the 3-gene clique to occur frequently in the bottom scenario but not in the top scenario. **(g)** A 4-gene module detected using FSM on brain data, **(h)** Gene ontology terms enriched in the 4-gene module of (g). (Criterion of selection: Fisher exact p-value < 0.03). **(i)** Histogram of “support” of all possible 4-gene cliques. Support refers to the number of cells where all pairwise relationships in the 4-gene set are significant by the PP test. The clique of (g) has a support of 72, far greater than all other cliques, **(j-k)** Example of two cells supporting the 4-gene module of (g). Each color represents a transcript of one of the four genes, grey represents all other transcripts.

InSTAnT provides two complementary routines for gene module discovery. The first routine, called Global Colocalization Clustering (GCC), identifies modules by representing the CPB test results as a matrix of gene-gene *d*-colocalization strengths and clustering rows and columns of this matrix (Methods). Figure 6a shows the results of such clustering for U2OS data, revealing two modules (top left) whose compositions are shown in Figure 6b. Module M1 (spatially illustrated in Figures 6d,e) consists of 14 genes, with 85 of 91 pairs being significantly *d*-colocalized and all but one of these significant gene pairs being assigned a perinuclear region annotation. Gene Ontology (GO) enrichment analysis of the module revealed shared annotations (p-value < 0.05, Figure 6c) related to cytoskeleton and ribonucleoprotein complexes. mRNA-cytoskeletal associations have been long known to play a key role in mRNA transport and targeting to specific subcellular locations, partly mediated by RBPs and ribonucleoprotein complexes^50,51^. Module M1 includes gene pairs whose protein products are known to interact, e.g., FASN-SPTBN1^52^ and PRPF8-SRRM2^53,54^. Module M1 also shares four genes with the nine-gene module called “Group II” found to colocalize (at a coarser resolution) in fibroblast MERFISH data^13^. The second module (M2) comprises eight genes, with 23 of 28 pairs being significantly *d*-colocalized, mostly with perinuclear annotation. The module is significantly enriched for several GO terms, e.g., positive regulation of cell death and receptor complex (Supplementary Figure 9), and its sub-cellular colocalization may thus mirror a co-functioning of its protein products.

A module reported by GCC comprises gene pairs whose *d*-colocalization is supported by many cells, but these supporting cells differ for different gene pairs and very few cells may have the entire module colocalized. Motivated by this, InSTAnT includes a second module discovery routine, called “Frequent Subgraph Mining” (FSM)^55^, that seeks a network of genes “colocalized” in many cells. (Colocalization of a network in a cell means that every edge in that network is a proximal gene pair in that cell (Figure 6f).) FSM can be used to find networks with a pre-specified minimum size (numbers of nodes and edges) that are supported by a large number of cells (Methods). For illustration, we used FSM to search for fully connected networks (“cliques”) with at least four genes and found a single module – *Sgk1, Ttyh2, Ndrg1* and *Ermn* (Figure 6g) – that is colocalized in 72 cells, far greater than the support of the next most frequent four-gene clique (12 cells) (Figures 6i–k). The six gene pairs comprising this module are *d*-colocalized individually, is specific to mature oligodendrocytes and the module is significantly associated (p-value 8.3e-3) with myelin sheath^56–59^ (Figure 6h). We speculate that their co-localization in specific partitions inside cell reflects coordinated transport and translation in mature oligodendrocytes.

## Discussions

In this work, we present the InSTAnT toolkit to screen for subcellular colocalization patterns of RNA pairs and modules in an unbiased manner, through rigorous statistical analysis of single-molecule resolution spatial transcriptomics data. We define *d*-colocalization as a new statistical phenomenon that may point to biological relationships such as RNA-RNA interactions, formation of condensates and shared subcellular localization. InSTAnT is a suite of statistical tests, at the heart of which lie the Proximal Pair (PP) test that finds colocalized gene pairs in a single cell and the Conditional Poisson Binomial (CPB) test that aggregates results of PP test across cells and reports *d-colocalized* gene pairs. InSTAnT provides spatial region annotations for the reported gene pairs to aid biological interpretation. It also includes procedures to characterize a *d*-colocalized gene pair based on its cell type specificity or spatial modulation and to identify colocalized gene modules.

We employed InSTAnT to detect hundreds of gene pairs with low false positive rate and high reproducibility on human U2OS cell line and mouse brain data. The InSTAnT analysis results suggest that *d*-colocalization map can provide insights into various types of molecular interactions: RNA-RNA interactions (Figure 3i), protein-protein interaction or shared pathway membership (Figure 3h) and RNA-protein interactions (Figure 3j–n). The RNA d-colocalized pairs can be used to infer detailed subcellular structures or characterize membrane-less organelles such as NS. These results indicate that the spatial distribution of RNAs has “texture” rather than being relatively random as previously perceived. Our brain data analysis shows that some RNA d-colocalized pairs have cell-type specificity, are spatially modulated, and share functional annotation with other colocalizing pairs. All these results suggest that RNA colocalization likely has biological consequences.

InSTAnT allows us to represent a cell as a graph where nodes represent genes and edges represent proximal gene pairs. Such a graph, along with the transcript count vector commonly used to represent an individual cell, may prove powerful in single cell analytics, allowing us to discover novel cell types through a more nuanced clustering of cells than possible using count vectors alone. It will be exciting to apply InSTAnT functionalities on future data sets that profile orders of magnitude more genes^26^ (~10K). There are straight-forward ways to adapt the toolkit to efficiently handle this scenario, such as by sampling of transcript pairs to estimate background probabilities in the PP test and by using a greedy approach to testing only a subset of gene pairs. We expect such applications to help us better characterize intracellular compartmentalization and provide complementary axes of information for discovering regulatory and signaling interactions with and between cells.

## Online Methods

### Code Availability

The code is available at https://github.com/anurendra/InSTAnT.

### InSTAnT user guide

InSTAnT tools have tunable parameters that can be selected based on the user’s requirement. We selected the scale parameter *d* based on the average cell’s diameter and threshold for CPB test based on False Positive Rate (1%) estimates. The user can also obtain region annotations of a gene pair’s colocalization if the data include masks for cell and nucleus boundaries. Similarly, they may run cell type specificity analysis if the data include cell type information. We advise caution when using InSTAnT with small distance thresholds, such as 1 μm or less, as the false positive rates in this regime can be high. This is due to the fact that colocalization with small distance is relatively rare in MERFISH data and the estimate of null probability of a pair of transcripts being proximal, a key aspect of the PP test, is error-prone in such cases. We believe that higher number of transcripts and improved optical resolution^17^ may alleviate this problem.

### U2OS Dataset

We obtained MERFISH data^32^ on a human osteosarcoma cell line (U2-OS) from http://zhuang.harvard.edu/MERFISHData/data_for_release.zip. We used the authors’ Matlab code to extract and output the data in table format. We filtered the data to retain transcripts having minimum area of 3 and intensity of 10^0.75^. The dataset had 7 replicates. We were able to extract data for four replicates – *rep2, rep3, rep4, rep5*; the other replicates presented severe memory management challenges and were not analyzed. Most of the reported results are from analysis of *rep3*, which profiles 130 genes in 3237 cells with an average of 1243 transcripts per cell. Global *d*-colocalization maps were constructed for all four replicates and compared to assess reproducibility.

### Brain Dataset

Data reported in Moffit et al.^37^ were obtained through personal communication with Dr. Jeffrey Moffitt. The dataset contained 6325 cells with 553 average number of transcripts across 7 z-planes. We obtained cell type assignment from Supplementary Table 1 from Moffit et al.^37^. We removed ambiguous cells leading to 5149 cells with 9 cell types. Proximal pairs were detected in cells that have at least one z-plane with 20 or more transcripts.

### MERFISH imaging and Analysis

#### General cell culture conditions:

U2 OS cells were cultured in minimal essential medium (MEM) from ATCC with 1 mM sodium pyruvate, 10% fetal bovine serum (FBS), and 1% penicillin-streptomycin (Pen-Strep). The cells were obtained from ATCC and maintained using the recommended protocol.

#### MERFISH sample preparation:

U2 OS MERFISH samples were prepared using a previously published method^60^. In brief, U2 OS cells were plated on a salinized 40mm #1.5 coverslip (Fisher Scientific). Plated cells were transferred to a 37 °C and 5% CO_2_ incubator overnight to grow. Cells were then fixed with 4% paraformaldehyde (Electron Microscopy Sciences) and permeabilized with 0.5% (vol/vol) Triton X-100 (Sigma Aldrich). Samples were stained with encoding probes (10nM/probe) and anchor probes (1μM) for 36 hours in a humidified incubator at 37 °C. To stabilize the cells during clearing, the stained cells were embedded in a thin, 4% polyacrylamide (PA) gel. Fiducial beads (Spherotech, FP-0245–2) were also included in the gel to align rounds of MERFISH images.

#### Commonly used imaging solutions:

The following solutions were used during imaging experiments described in this work. Readout wash buffer was adapted from Moffit et al.^60^ and contained 10% (v/v) ethylene carbonate (Sigma Aldrich), 0.1% Triton X-100 in 2x SSC. Imaging buffer adapted from Moffit et al.^60^ and contained 5mM 3,4-dihydroxybenzoic acid (PCA; Sigma Aldrich), 2 mM trolox (Sigma Aldrich), 50 μM trolox quinone, 1:500 of recombinant protocatechuate 3,4-dioxygenase (rPCO; OYC Americas), adjusted to a pH of 7–7.2 using 1 N NaOH (VWR International) in 2x SSC. Cleavage buffer was adapted from^60^ and contained 0.05 M TCEP HCl, adjusted to a pH of 7–7.2 using 1 N NaOH, in 2x SSC. Stripping buffer was adapted from Eng. et al.^14^ and contained 55% formamide, and 0.1% Triton X-100 in 2x SSC.

#### MERFISH imaging:

All images were acquired using a Zeiss Axiovert-200m widefield microscope (Carl Zeiss AG) located in the IGB core imaging facility. The sample was placed into a flow cell (Bioptechs, FCS2), filled with RNAse free 2x SSC, and connected to a lab built automated flow system. Briefly, computer-controlled valves (Hamilton, MVP/4, 8–5 valve) are used to select which solution was pulled across the sample by a computer controlled pump (Gilson, Minipuls 3). All systems are controlled by a custom designed Python script that can communicate with the microscope to start imaging or start flowing after an imaging round is done. In brief, a single round of imaging involves staining with fluorescently labeled readout probes (0.4 mL/min for 6 minutes, and 0.34 mL/min for 6 minutes), washing with readout wash buffer (0.23 mL/minute for 9 minutes) to remove unbound probes, and imaging buffer was flowed into the flow cell prior to imaging (0.34 mL/minutes for 6 minutes) to reduce photobleaching. A single quad band excitation filter (Chroma, ZET402/468/555/638x) and dichroic (Chroma, ZT405/470/555/640rpc-UF1) were used to image all samples. Excitation was provided by a 7 laser system (LDI WF, 89 North). Alexa Fluor 647 (Fisher scientific) labeled probes were excited using a 647 nm laser (0.5 W) with a ET700/75m (Chroma) emission filter, and 1.5 second exposure time. Atto 565 (Atto tec) labeled probes were excited using a 555 nm laser (1 W) with a ET610/75m (Chroma) emission filter, and a 0.75 second exposure time. Fiducial beads were imaged with a 405 nm laser (0.3 W) with a ET440/40m emission filter, and a 1 second exposure time. Samples were imaged with a 63x oil immersion objective (Carl Zeiss AG, 420782-9900-000), and focus was maintained between imaging rounds using Definite Focus (Carl Zeiss AG). 9 z planes with 0.7 μm steps were taken for each FOV, and a total of 100 FOVs were acquired. After imaging is complete, a cleavage buffer (0.2 mL/minute for 15 minutes) was flowed across the sample to remove the fluorophores from the probes. The cleavage buffer was washed away using RNAse free 2x SSC (0.5 mL/minute for 10 minutes). This process was repeated for a total of 8 rounds of imaging. PolyA probes were stained after the final imaging round using the same method as described above.

#### MERFISH data processing:

Individual FOVs were exported from czi format into 16 bit tiff format using Zen (Carl Zeiss AG) using the image export method. Images then were reformatted into image stacks by FOV and round. A modified copy of MERLIN^61^ was used to decode MERFISH spots. In brief, for each FOV, images from different rounds are aligned using fiducial beads that were imaged in each round. Aligned images are then normalized, decoded, and identified spots filtered using previously published methods^26^. Cell segmentation was done separately from MERLIN using Cellpose^62^ on PolyA and DAPI images for each FOV. To improve FOV alignment to neighboring FOVs, the DAPI channel was used with the restitching function found in Zen (Edge detection: on, minimal overlap: 5%, maximal shift: 15%, comparer: best, Global optimizer: best). Using the aligned images, segmented cells that cross FOV boundaries were merged into single cells, and global positions were generated for each spot. Spots are then assigned to cells based on their spatial coordinates. Spots were then filtered to remove any spot smaller than 3 pixels in size.

#### smFISH probe design:

All smFISH probes were designed using the Stellaris probe designer (Biosearch technologies). Probes were designed using the following settings: Masking level: 5, max number of probes: 48, oligo length: 20, minimum spacing length: 2. SRRM2 exon probes were designed against SRRM2 isoform ENST00000301740 (GRCh38.p13). SRRM2 intron probes were randomly selected from probes designed for three different introns defined by ensemble (SRRM2–230 intron 1, SRRM2–230 intron 2, and SRRM2–230 intron 10) (GRCh38.p13). MALAT1 probes were designed against MALAT1 isoform ENST00000534336 (GRCh38.p13). All probes were purchased from Biosearch modified with mdC (TEG-Amino) at the 3’ terminus. The probes were dissolved in TE buffer and labeled using AF488/Cy3/Cy5 NHS esters for MALAT1, SRRM2 intron, and SRRM2 exon, respectively. The labeled probes were purified using the Bio-Rad Bio-Spin P-6 purification columns (Cat # 732–6221).

#### smFISH sample preparation:

Approximately 1.5–1.8 million U2OS cells were plated on a #1.5, 40 mm coverslip (Fisher Scientific) that has been UV treated before plating. The cells were then transferred to an incubator at 37 °C and 5% CO2, overnight for 12–16 hours.

Modified from Fei et al.^63^, the sample was rinsed with 1x PBS (Corning), followed by fixation using 4% paraformaldehyde (PFA; Electron Microscopy Sciences) in 1x PBS for 10 minutes at room temperature (RT). The sample was then washed three times with 1x PBS and permeabilized with 0.5% Triton X-100 (Sigma Aldrich), 2 mM vanadyl ribonucleoside complexes (VRC; Sigma Aldrich) in 1x PBS for 10 minutes on ice, followed by three quick washes with 1x PBS. At this point, the sample can be stored in 70% Ethanol at 4 °C if the experiment needs to be paused temporarily.

To prepare for smFISH hybridization, sample was rinsed with 10% formamide (Sigma Aldrich) in 2x saline sodium citrate (SSC; Fisher Scientific). smFISH probe hybridization buffer was prepared with 0.2 mg/mL of bovine serum albumin (BSA; Fisher Scientific), 2 mM VRC, 10% dextran sulfate (Sigma Aldrich), 1 mg/mL yeast tRNA (Fisher Scientific), 10% formamide, 1% murine RNase inhibitor (New England BioLabs) in 2x SSC. Avoid light exposure from this point forward. smFISH probes were then added to the FISH hybridization buffer at a final concentration of 14 nM for each targeted RNA (MALAT1, SRRM2 intron, and SRRM2 exon).

A humidified chamber was made using an empty pipette box filled halfway with nuclease-free water (Corning) at the base and a UV-treated glass slide covered with a parafilm layer on top. A 100 μl drop of the FISH probe hybridization buffer was then added on top of the parafilm layer and the sample was casted over the drop with the cell side facing down. The chamber was then placed in an incubator in dark and wrapped entirely with aluminum foil overnight at 37 °C for at least 16 hours. The sample was quickly rinsed two times with 10% formamide in 2x SSC then stained with 4’,6-diamidino-2-phenylindole (DAPI; Invitrogen by Fisher Scientific) 1:1000 of 1 mg/mL stock solution and 1:5000 of Fluoro-Max Blue Aqueous Fluorescent Particles (fluorescent beads; Fisher Scientific) in 2x SSC. The sample was incubated with the DAPI and fluorescent beads solution for 5 minutes while rocking at RT, followed by a quick wash with 2x SSC, then stored in 2x SSC at 4 °C until ready for imaging.

#### Protein staining:

After smFISH imaging, the sample can be stored in 1x PBS at 4 °C for up to a week before protein staining. Samples were fixed a second time with 4% PFA in 1x PBS for 5 minutes at RT, then rinsed three times with 1x PBS. This was followed by incubation with a blocking solution of 1% BSA in 1x PBS for three consecutive times with 10 minutes each time at RT.

The SON primary antibody (Anti-SON, Sigma Aldrich, HPA023535) was kept at −20 °C until ready for use. The primary antibody stock solution of 1:1000 was prepared with 1x PBS and kept on ice. A 1:5000 primary antibody dilution was prepared in blocking solution and the sample was incubated with 200 μl of the primary antibody solution for approximately 1 hour at RT in the dark.

The sample was washed with blocking solution three consecutive times with a 10-minute incubation each time at RT, followed by three washes with 1x PBS, for 10 minutes each time at RT.

Secondary antibody was conjugated to Alexa Fluor 647 (Goat anti-rabbit, Invitrogen, A21245). The concentrated secondary antibody was kept at 4 °C until ready for use. Sample staining was accomplished by 1:1000 dilution of the secondary antibody in blocking solution and casting of the sample on a 200 μl drop of the secondary antibody solution, with the cell side facing down. The sample was then incubated for 1 hour in the dark at RT. The sample was re-stained with DAPI in 1x PBS with the same concentration and incubation time described in smFISH staining section. This was followed by a quick rinse with 1x PBS and the sample was stored in 1x PBS at 4 °C until ready for imaging.

#### smFISH image acquisition:

smFISH and protein imaging were done on the same MERFISH imaging and fluidic system described above (MERFISH imaging). After placing the sample into the flow cell, imaging buffer was flowed through the system (0.34 mL/minute for 5 minutes). Excitation and dichroic filters were the same as used above. The following dyes, lasers, and emission filters were used for smFISH imaging.

**Table:** 

Channel	Target	Laser line (power)	Exposure time	Emission filter
DAPI	Fiducial beads, nuclei	405 nm (0.3 W)	0.075 seconds	ET440/40m
Alexa Fluor 488	MALAT1 lncRNA	470 nm (1 W)	2 seconds	ET525/50m
Cy3	SRRM2 intron RNA	555 nm (1 W)	2 seconds	ET610/75m
Cy5	SRRM2 exon mRNA	640 nm (0.5 W)	3 seconds	ET700/75m

Samples were imaged with the same 63x oil immersion objective as above, and focus was maintained between imaging rounds using Definite Focus. 9 z planes were imaged with a step size of 0.7 μm. After imaging, smFISH probes were removed using a stripping buffer that was flowed through the system (0.34 mL/minutes for 5 minutes) without removing the sample from the microscope. After stripping the sample was washed with 2x SSC (0.5 mL/minutes for 5 minutes). The sample was imaged a second time using the same settings as above. After imaging the sample was removed from the flow cell and placed into 1x PBS prior to protein staining (Protein staining).

After protein staining was complete, sample was placed into the flow cell and filled with imaging buffer. The same region imaged during the smFISH experiment was found and reimaged using the same objective and z stack settings as above. The following imaging settings were used.

**Table:** 

Channel	Target	Laser line (power)	Exposure time	Emission filter
DAPI	Fiducial beads, nuclei	405 nm (0.3 W)	0.05 seconds	ET440/40m
Alexa Fluor 647	SON protein	640 nm (0.5 W)	1.5 seconds	ET700/75m

#### SRRM2 image registration and alignment:

Individual FOVs were exported from czi format into 16 bit tiff format using Zen’s (Carl Zeiss AG) image export method. To align images from the same FOV across multiple rounds of imaging or experiment, blue fluorescent beads imaged in the DAPI channel were used as fiducial markers. We found that aligning images from the same experiment required a simple translation. To align protein images with mRNA images, an iterative rotation and translation process was developed. For each iterative round of alignment, the protein DAPI channel was rotated, then translated to best align with the mRNA image, this warped image was then used as the starting protein DAPI image for the next round of alignment. We found that it took between 2 and 5 rounds of alignment to align protein images to mRNA images. Chromatic aberration was corrected by aligning all channels to the Cy5 channel. Multicolor beads (Multi-speck bead slide, Carl Zeiss AG, 1783–455) that included dyes in the Alexa Fluor 488, Cy3, and Cy5 channels were used to correct Alexa Fluor 488 and Cy3 channels. The DAPI channel was corrected to the Cy5 channel using the fiducial bead cross talk between the DAPI and Alexa Fluor 488 channels. This was done by calculating the shift between non-nuclear regions of the DAPI and Alexa Fluor 488 channels, then adding the Alexa Fluor 488 to Cy5 shift to the DAPI to Alexa Fluor 488 shift.

#### SRRM2 image preprocessing:

To remove cross talk in DAPI and Alexa Fluor 488 channels caused by the fiducial beads, stripped Alexa Fluor 488 mRNA channel was subtracted from the stained Alexa Fluor 488 channel. As fiducial beads are not affected by the mRNA stripping conditions, any spots that remain in the stripped Alexa Fluor 488 channel would be from the beads, not from MALAT1 mRNA. In order to reduce background in other images, round subtraction was also done on the other channels of the mRNA FOV.

#### SRRM2 co-localization analysis:

Co-localization analysis was done on a single z plane from each experiment stack. Images were then filtered using a high pass filter (5 pixel sigma) and Lucy–Richardson deconvolution (10 iterations, 9 pixel filter size, 1.4 pixel sigma). Filtered images are then converted to binary masks with manually defined thresholds. To remove false positives in the MALAT1 channel, the MALAT1 mask was multiplied with the inverse of the stripped MALAT1 mask. Cell nuclei were identified using the DAPI channel, and segmented using a manually defined threshold.

The co-localization rate was calculated for each nucleus defined from the DAPI channel. To calculate the co-localization rate between two channels, each channel is multiplied against the nuclei mask. For each spot in the first mask, the spot was dilated by 2 μm and then compared against the second mask. If the dilated spot overlaps any spot in the second mask, it is considered to be colocalized. The colocalization rate was then calculated to be the following:

$$\text{colocalization percent}=\frac{\text{Co}-\text{localized spots ct}}{\text{Total spots ct}}*100\text{\%}$$

The colocalization percent was averaged across 13 cells.

#### SRRM2 figure and generation (Figures 3k–n):

SRRM2 exon and intron images were filtered using a high pass filter with 2 pixel sigma, while MALAT1 was filtered using high pass filter with 5 pixel sigma. Raw SON images were used in panels Figures 3 m,n.

### False Positive Rate (FPR)

We generate random baseline dataset established by permuting the gene labels of all transcripts within each cell, which recapitulates the spatial patterns of the original data but not the gene-gene relationships. FPR is obtained by comparing the number of detected pairs obtained on randomized data with number of detected pairs on real data.

Ten of the 140 genes probed in the U2OS MERFISH data set were “blanks”, meaning that they do not represent any particular RNA or other molecule. Any gene pair involving such blank “genes”, if found to d-colocalize, is clearly a false positive. This provided us another opportunity to assess the false positive errors in our global co-localization map. We recorded the fraction of such false positives among predicted pairs at varying levels of significance (Supplement Figure 2, blue).

### Hyperparameter selection

Scale parameter d was chosen to be 4 microns in U2OS dataset and 2 microns for Brain dataset, as it corresponded to ~5% of average diameter of a cell in the respective datasets. The p-value threshold for PP test was chosen to be 0.01 for both the datasets which resulted in FPR~5%. p-value threshold for CPB test was chosen to be 1e-3 for U2OS and 1e-5 for Brain dataset as it resulted in FPR<1%. p-value threshold for frequent subgraph mining on Brain dataset was chosen to be 0.05 as threshold of 0.01 didn’t yield any subgraph.

### Proximal Pair (PP) test

PP test reports proximal pairs of genes in a particular cell. A gene pair *g*_*i*_, *g*_*j*_ is a proximal pair in a cell if their transcripts are proximally located (separated by distance *d* or less) significantly more often than expected by chance. The null probability *p* is estimated from the distances between all pairs of transcripts (regardless of gene identities) in the cell, by calculating the fraction of transcript pairs that are proximally located. Let *t*_*i*_ and *t*_*j*_ denote the transcript counts of genes *g*_*i*_, *g*_*j*_ respectively in the cell, let *T* = *t*_*i*_*t*_*j*_ and let *K* be the number of proximally located transcript pairs of these genes. The PP test performs a Binomial test providing a p-value for *g*_*i*_, *g*_*j*_ as

$$\text{p-value}\left(g_{i},g_{j}\right)=\text{Binomial }(T,p,K)$$

### PP-3D test

PP-3D is an extension of PP test to handle three-dimensional data in the form of 2D (x-y) locations of transcripts in each of multiple *z*-planes. We assume that data from different planes are independent and identically distributed. The new distribution is the sum of independent Binomial distributions (with the same parameter), which is also a Binomial distribution. The null probability of two transcripts being proximal is estimated as a weighted combination of estimated null probability for each of the z-planes,

$$p\equiv \frac{\sum_{z}l_{z}p_{z}}{\sum_{z}l_{z}}$$

where, *p*_*z*_ denotes the null probability for *z*-th plane, *l*_*z*_ denotes the total number of transcripts in *z*-th slice. *T* and *K* are also aggregated across z-planes:

$$T=\underset{z}{\sum }T_{z}$$

$$K=\underset{z}{\sum }K_{z}$$

where *K*_*z*_ is total number of proximal transcript pairs and *T*_*z*_ is total number of transcript pairs (of *g*_*i*_, *g*_*j*_) in *z*-th plane. PP-3D calculates a p-value for each gene pair as p-value *g*_*i*_, *g*_*j*_ = *Binomial* (*T*, *p*, *K*).

### Conditional Poisson Binomial (CPB) test

CPB test detects a *d*-colocalized gene pair, i.e., a gene pair that is a proximal pair in significantly many cells. It assigns a p-value to the number of cells in which a gene pair is found to be proximal pair detected using PP test. We first describe a simpler version of the test (“unconditional Poisson Binomial” or UPB) test that assumes that all gene pairs are equally likely to be proximal pair in a cell but allows for the fact that different cells may have different number of proximal pairs. Let $X_{ij}^{c}$ be a binary variable denoting if *g*_*i*_, *g*_*j*_ are a proximal pair in *c*-th cell. $X_{ij}^{c}$ is assumed to follow a Bernoulli distribution with parameter $p_{0}^{c}$, which is estimated as the fraction of proximal gene pairs in the cell:

$$p_{0}^{c}=\equiv \frac{\sum_{k\leq l}X_{k,l}^{c}}{\sum_{k\leq l}1}=\frac{\sum_{k\leq l}X_{k,l}^{c}}{\left(\begin{array}{l} n\\ 2 \end{array}\right)}$$

where *n* denotes total number of genes. This estimate of $p_{0}^{c}$ assumes that all gene pairs can be a proximal pair. To incorporate the fact that a gene pair cannot be a proximal pair if either of the genes is not expressed in the cell, the above estimate is modified as,

$$p_{0}^{c}\equiv \frac{\sum_{k\leq l}X_{k,l}^{c}}{\sum I_{k\leq l}\left(g_{k},g_{l}\right)}$$

where *I*(*g*_*k*_, *g*_*l*_) is an indicator function that equals to 1 iff both *g*_*k*_ and *g*_*l*_ are expressed.

CPB test is a modified version of the UPB test that accounts for the possibility that all gene pairs are not equally likely to be colocalized in a cell and sets the Bernoulli parameter ($p_{0}^{c}$ above) to be gene pairdependent. Let *z*_*i*_ denote total number of proximal pairs having gene *i* as one of the genes, aggregated across all cells, i.e.,

$$Z_{i}=\sum_{j\leq c}X_{ij}^{c}$$

We use these global summary statistics to model the prior probability Π_*ij*_ that a proximal pair detected in a cell is the gene pair *g*_*i*_, *g*_*j*_, as follows:

$$\Pi_{ij}\equiv \frac{z_{i}z_{j}}{\sum_{i\leq j}z_{i}z_{j}}$$

This model de-emphasizes gene pairs comprising genes that are frequently found to be in proximal pairs across cells. Now, the Bernoulli parameter for variable $X_{ij}^{c}$ is estimated as

$$p_{ij}^{c}\equiv 1-{\left(1-\Pi_{ij}\right)}^{\sum_{i\leq j}X_{ij}^{c}}$$

The total number of cells where *g*_*i*_, *g*_*j*_ is a proximal pair follows a Poisson Binomial distribution

$$\overset{m}{\underset{c=1}{\sum }}X_{ij}^{c}\sim \text{Poisson Binomial }\left(p_{ij}^{1},\ldots ,p_{ij}^{m}\right)$$

### Spatial Annotation

A d-colocalized pair is annotated by cellular region where the gene pair’s proximal pairs tend to be found. We define four categories – Nucleus (Nuc), Peri-Nucleus (PN), Cytosol (Cyto) and Cell Periphery (CP). Proximal pairs in each cell are annotated by cellular region and is aggregated across cells to yield primary and secondary category. Perinuclear (PN) region is defined as including x microns on either side of the nuclear membrane, while Cell Periphery (CP) is defined as regions within y microns of the cell membrane. Remaining regions are designated as Cytosol (Cyt) or Nucleus (Nuc). We chose x = 2.5 micron which corresponded to ~43% of nucleus transcripts being annotated as perinuclear, and y = 4 micron which corresponds to ~35% cytosolic transcripts being annotated as cell periphery.

### RNA-RNA Interaction (RRI)

For RRI, we set distance *d* to be equal to the resolution of MERFISH data (200 nm). The small distance was chosen to capture gene pairs whose *d*-colocalization may be explained due to the binding of their transcripts. We used RNAplex^34^ to compute the RRI scores. For this, we retrieved the nucleotide sequences from the Ensembl database^64^ and got the specific transcript id to get the correct spliced form. RNAplex has been shown to be among the most accurate tools while being fast enough to compute the scores for gene pairs with their full transcripts. Finally, we perform a gene-centric analysis for each of the 130 genes. For each gene, we ask if top 10 *d*-colocalized pairs (out of 130) has significantly higher number of pairs with RRI score greater than a fixed threshold (RRI>35). We perform a Binomial test whose success probability is obtained as follows. We model background distribution by fitting a Gaussian distribution to the RRI scores of the pairs with *d*-colocalization score greater than 0.01. The survival probability of RRI scores higher than the fixed threshold (RRI>35) serves as the success probability of Binomial test. Finally, we perform an FDR correction using the Benjamini-Hochberg procedure^65^. 8 of the genes pass this FDR correction showing that RRI may be a plausible mechanism for their d-colocalized pairs.

### Enrichment Analysis

To understand the biological mechanism or consequences of d-colocalization, we tested if the compendium of d-colocalized gene pairs has significant overlap with functionally related gene pairs. We define a gene pair to be functionally related if both genes are present in same KEGG pathway or are annotated with same GO terms more than K times. K was chosen such that number of gene pairs is similar across d-colocalized and functionally related set. In our analysis, K (MF) = 2, K (BP) =1, K (CC) = 3, K (pathway) = 1. We performed a hypergeometric test between d-colocalized pairs and functionally related set.

### Cell Type Specificity of a *d*-colocalized Gene Pair

InSTAnT employs a series of statistical tests to categorize a d-colocalized pair based on its cell type specificity. First, it tests the association between cells where a gene pair was deemed a significant proximal pair and cells of a particular type (e.g., inhibitory neurons), using a Hypergeometric test. (This process is repeated for every cell type.) If such an association is found to be statistically significant, it is subjected to further tests to determine if the cell type specificity arises simply because one of the genes in the pair is expressed specifically in that cell type. For this, InSTAnT utilizes a version of the generalized Hypergeometric test that tests for an association between two sets conditional on a third set^66^, as described below. In this case, the third set comprises the cells with high expression of one of the genes in the pair.

Let *U* be the set of all cells, *M* be the set of cells of a particular cell type, *O* be the set of cells where a gene pair is deemed a proximal pair and *E* be the set of cells with high expression of one of the genes in the pair. *M*, *O* and *E* are subsets of *U*. The threshold for high gene expression used in defining *E* is chosen such that size (*E*) = size (*M*). Let |*M* ∩ *E|* = *γ*, |*M* ∩ *O*| = λ, |*E* ∩ *O| = α|*. The Hypergeometric test p-value of association between *M* and *O* is given by the probability that a random set of size |*O*| has an overlap (intersection) of size greater than or equal to *λ* with *M*. However, we wish to test if the overlap between *M* and *O* is significant beyond what is expected not from a random set of size |*O*| but a random set of this size that respects the known overlap between *M* and *E* and between *E* and *O*. For this, we calculate probability of the overlap between *M* and a random set of |*O*| being greater than or equal to *λ* conditional on the observed overlap between *M* and *E* and that between *E* and *O*, as follows:

$$\frac{\sum_{k=\lambda }^{\text{min}(|M|,|O|)}\sum_{\beta =0}^{k}\left(\begin{array}{c} \gamma \\ \beta \end{array}\right)\left(\begin{array}{c} m-\gamma \\ k-\beta \end{array}\right)\left(\begin{array}{c} n_{1}-\gamma \\ \alpha -\beta \end{array}\right)\left(\begin{array}{c} |U|-|M|-|E|+\gamma \\ |O|-\alpha -k+\beta \end{array}\right)}{\left(\begin{array}{c} \left|E\right|\\ \alpha \end{array}\right)\left(\begin{array}{c} \left|U|-|E\right|\\ |O|-\alpha \end{array}\right)}$$

This is an example of multivariate hypergeometric distribution. We use *scipy.stats.multivariate_hypergeom* package for multivariate hypergeometric distribution.

For each gene pair that is associated with a cell type, InSTAnT performs the above test twice, each time conditioning on a set *E* defined by the high expression cells for one of the genes of the pair. Significant p-values in both tests thus performed indicate that the cell type-specificity of the d-colocalized gene pair is significant beyond what is expected from the specificity of either gene’s expression. Furthermore, InSTAnT tests if either gene of the pair is a marker of the cell type, defined as any gene among the top 10 by association between their expression and the cell type. A marker gene is found by conducting Hypergeometric test of overlap between *O* and *E*.

Using the above tests, InSTAnT categorizes a *d*-colocalized gene pair vis-à-vis its cell type specificity as follows: If the gene pair is significantly associated with a cell type (first test above), then it belongs to Category 1 if the association is significant by the Hypergeometric test conditional on high expression cells of both genes and neither gene is a marker of the cell type, otherwise it belongs to Category 2. Category 3 comprises *d*-colocalized gene pairs that are not associated with any cell type (Bonferroni corrected hypergeometric p-value >= 0.05, Supplement Table 5).

### Probabilistic graphical model for Spatial Modulation

InSTAnT uses a likelihood ratio test to determine if sub-cellular colocalization of a *d*-colocalized gene pair is spatially modulated at the tissue level. Informally, this means that the cells in which the gene pair is deemed to be a proximal pair are non-randomly distributed in the physical space.

The probabilistic model is formulated around a graph with a node for each cell and edges between neighboring cells. Two cells are neighboring cells if they are located within a configurable distance (set to 100 micron in our tests). Each node is associated with a binary variable *s*_*c*_ that indicates whether the specific gene pair (say *g*_*i*_, *g*_*j*_) is a proximal pair in the corresponding cell *c*, as detected by the PP test. The variable *s*_*c*_ is assumed to be a Bernoulli-distributed variable. The null hypothesis is that the Bernoulli parameter is a global constant *p*^*global*^ shared across all cells, i.e., it does not depend on the cell *c* and thus on its spatial location:

$$H_{0}:s_{c}\sim Ber\left(p^{\text{global}}\right)$$

*p*_*global*_ is estimated as the fraction of cells where the gene pair *g*_*i*_, *g*_*j*_ is a proximal pair, which is its maximum likelihood estimate. In the alternative hypothesis, the model assumes that the distribution of variable *s*_*c*_ depends on the fraction of cells *c*′ in the neighborhood of *c* for which *s*_*c*_ = 1. Let *p*^*local*^ be the fraction of cells *c*^′^ in the neighborhood of *c* for which *s*_*c*_ = 1.

$$\begin{array}{c} H_{1}:s_{c}\sim Ber\left(w\;p^{\text{local}}+(1-w)p^{\text{global}}\right)\\ 0<w<1 \end{array}$$

The parameters *p*^*global*^, *p*^*local*^, *w* are learnt by maximizing likelihood. Weight *w* controls the contribution of local neighborhood. InSTAnT calculates the log likelihood ratio (LLR) for each gene pair in the *d*-colocalization map and pairs with LLR above a threshold are designated as spatially modulated. The threshold is obtained by random permutation of the of *s*_*c*_ values of cells, repeating the above test and selecting the highest LLR score (over all gene pairs) seen on the randomized data. This allows us to detect spatially clustered distributions of cells supporting *g*_*i*_, *g*_*j*_ colocalization.

### Module Discovery: Global Colocalization Clustering (GCC)

GCC is a procedure to analyze a *d*-colocalization map to identify subsets of genes that exhibit a high frequency of pairwise *d*-colocalization relationships. To this end, it represents the *d*-colocalization map as an *n* x *n* matrix (*n* = number of genes) whose entries are the negative logarithm of p-values of gene pairs from the CPB test and performs a hierarchical clustering of rows and columns using Euclidean distance with Ward criterion. (The constant 1e-64 is added to all the p-values to handle zero p-values prior to taking logarithms.)

### Module Discovery: Frequent subgraph mining (FSM)

FSM seeks a network of genes that is “colocalized” in many cells, where colocalization of a network in an individual cell means that every gene pair connected by an edge in that network is a proximal pair in that cell. It constructs a *colocalization graph* for each cell with genes as nodes and edges representing proximal gene pairs from PP test. It then uses an efficient graph mining tool called gSPAN^55^ to detect subgraphs with a pre-specified minimum size (numbers of nodes and edges) that are supported by a pre-specified minimum number of cells.

## Supplementary Material

Supplementary Figures
