# Supplementary material for: Intracellular Spatial Transcriptomic Analysis Toolkit (InSTAnT)
Source: Res Sq. 2023 Jan 27:rs.3.rs-2481749. Preprint. [Version 1] doi: 10.21203/rs.3.rs-2481749/v1 (PMC9901031; doi:10.21203/rs.3.rs-2481749/v1)
Supplement: Supplementary Figures [file SupplementaryFigures.pdf]

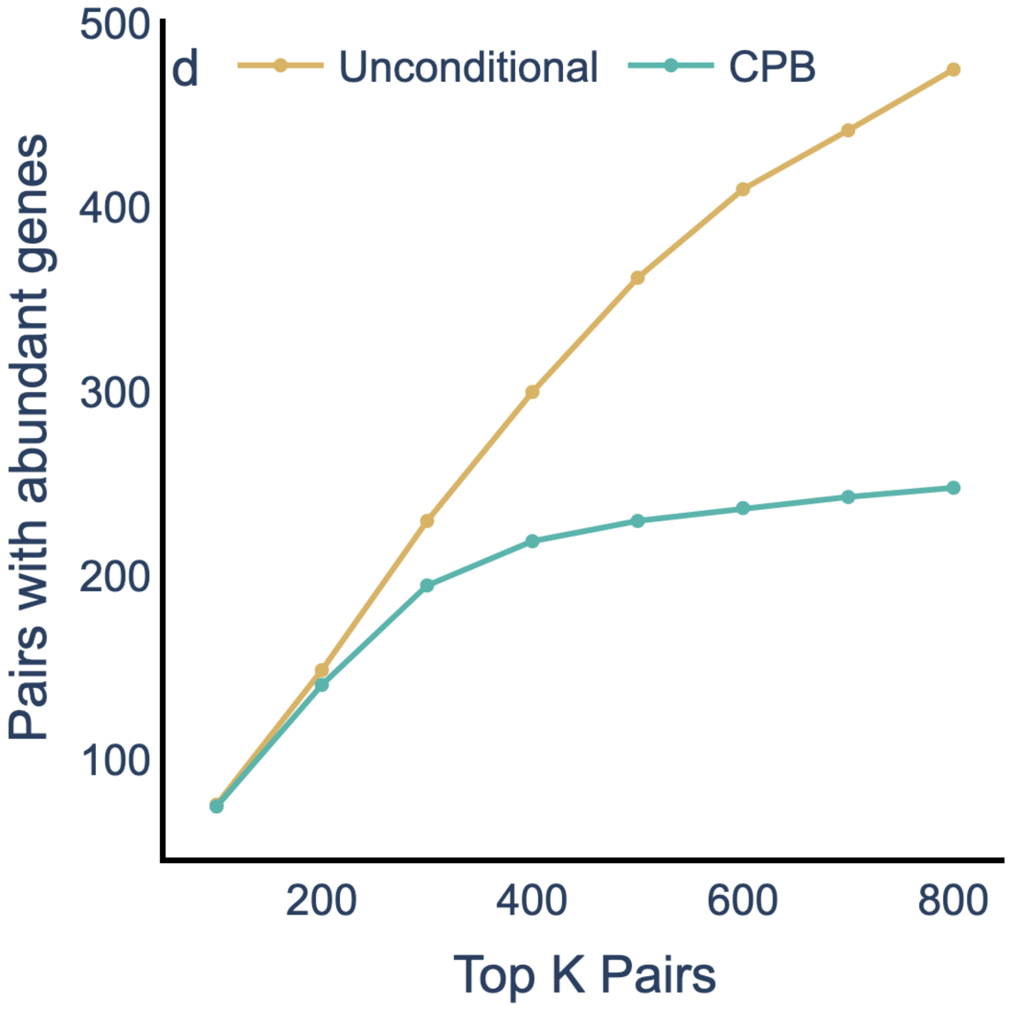

Supplementary Figure 1

Conditional Poisson Binomial (CPB) test negates the bias that may be due to individual gene’s expression or specific intracellular localization. The null probability in CPB test is conditioned on global summary statistic of each of the individual genes. An abundant gene is defined as 5 highest expressed genes across cells.

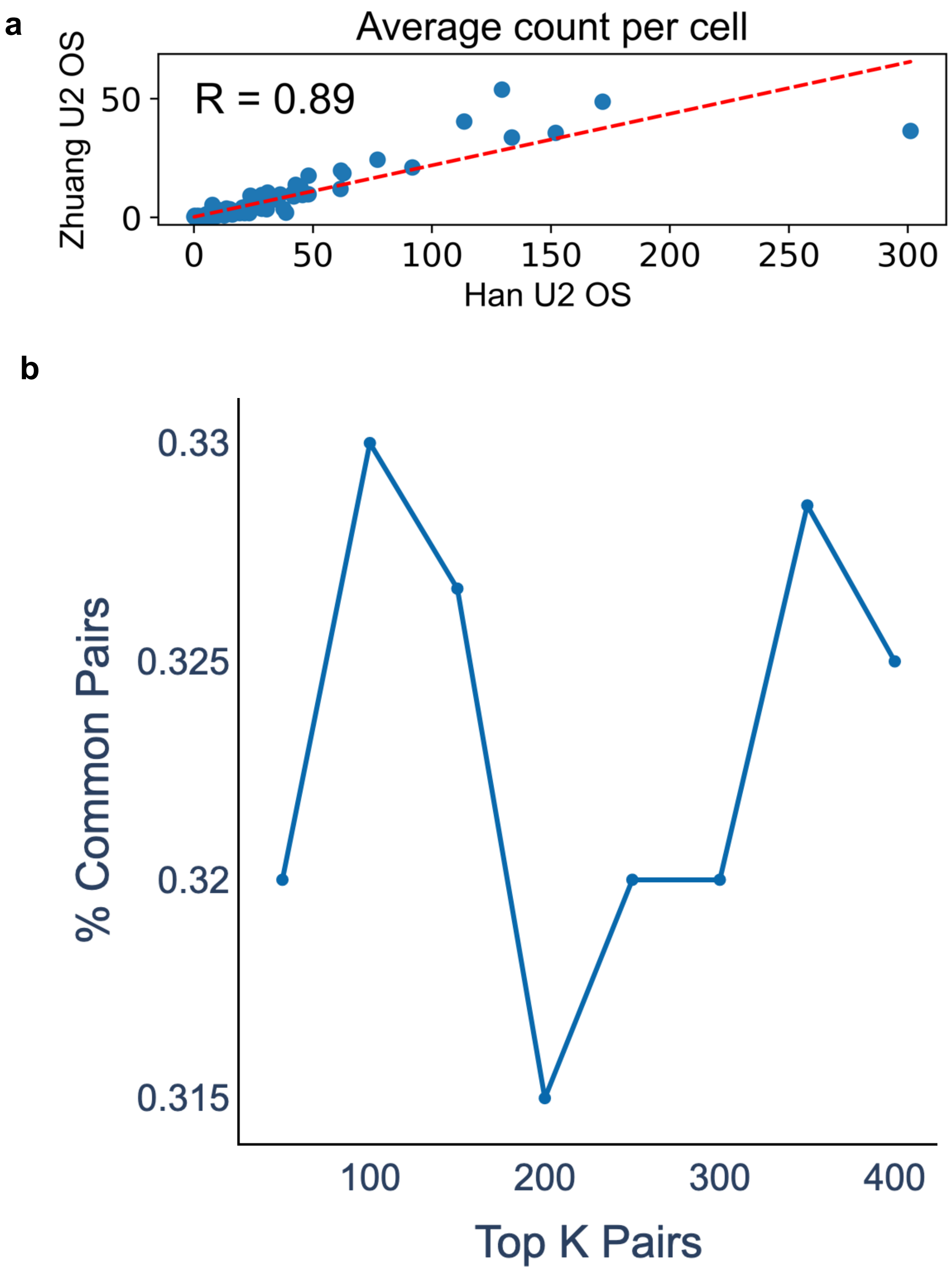

Supplementary Figure 2

2(b) shows that ~30% of top correlated gene pairs overlap across Zhuang and Han U2OS data. 2(a) shows high reproducibility across datasets if we perform gene level analysis.

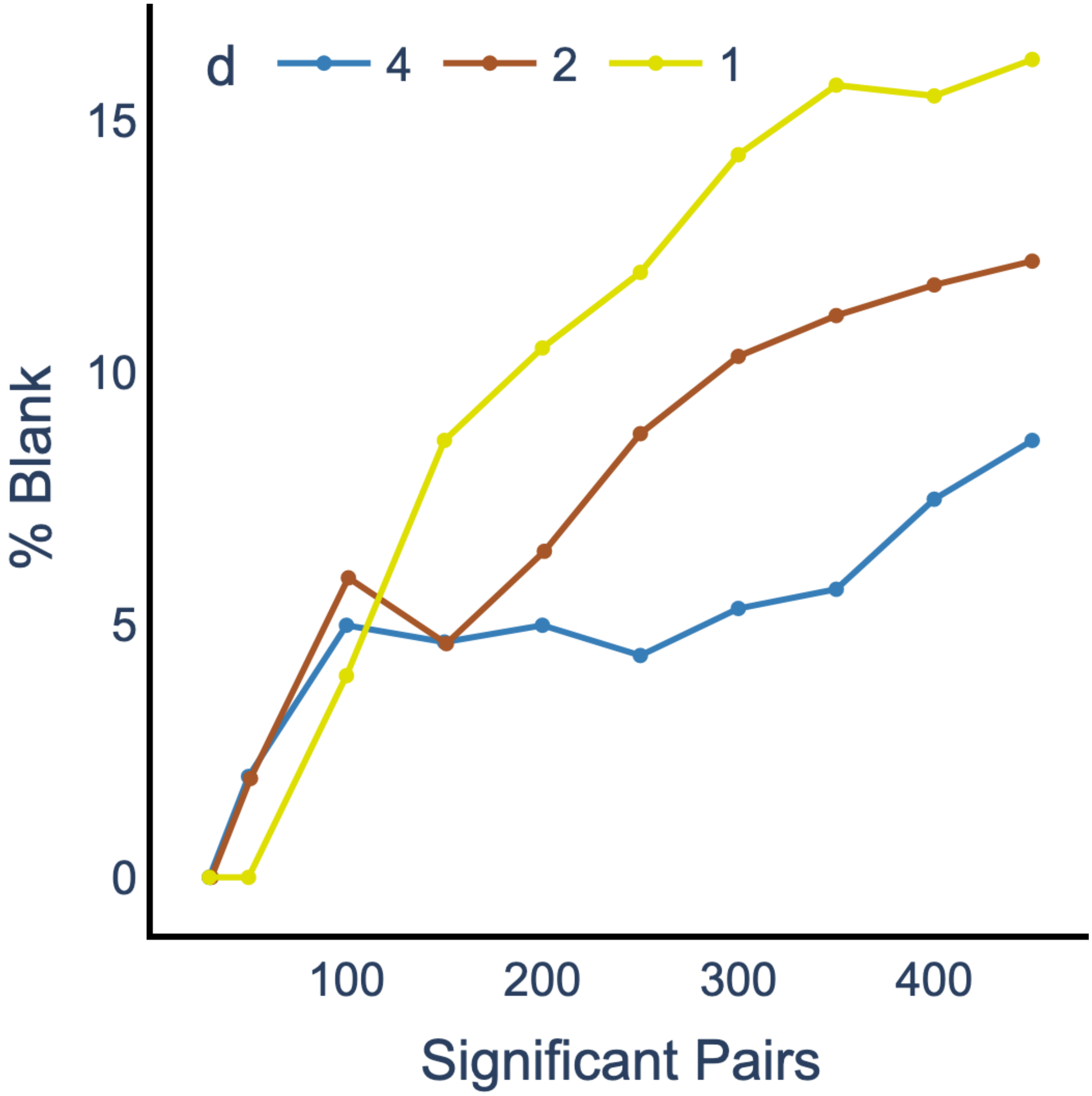

Supplementary Figure 3

FPR estimated as the fraction of significant gene pairs (by CPB test) that include at least one blank “gene”, at varying p-value thresholds. InSTAnT predicts ~400 d-colocalized pairs at d = 4 with low blank fraction (~5%)

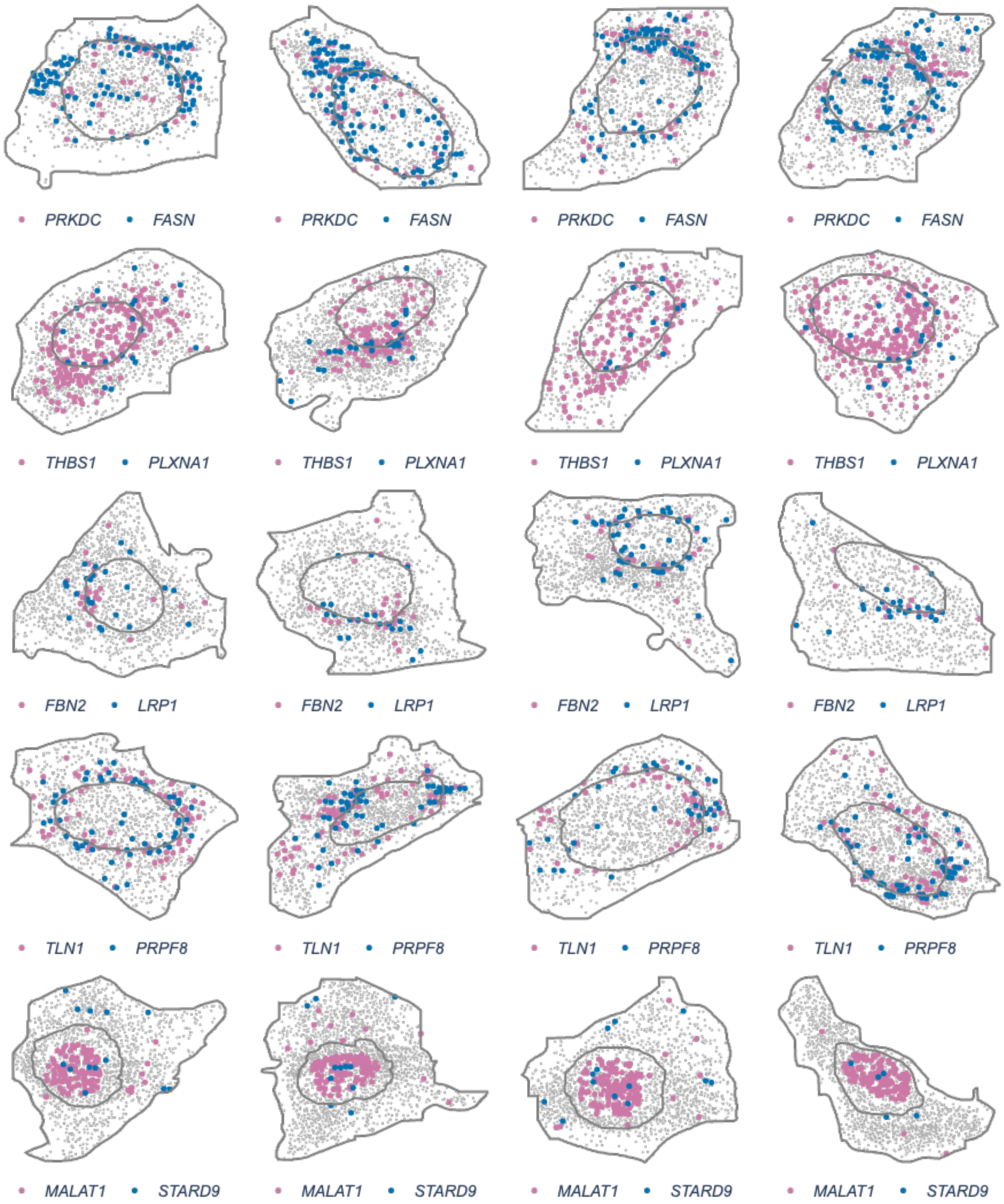

Supplementary Figure 4

Visualization of a few d-colocalized pairs in sample cells.

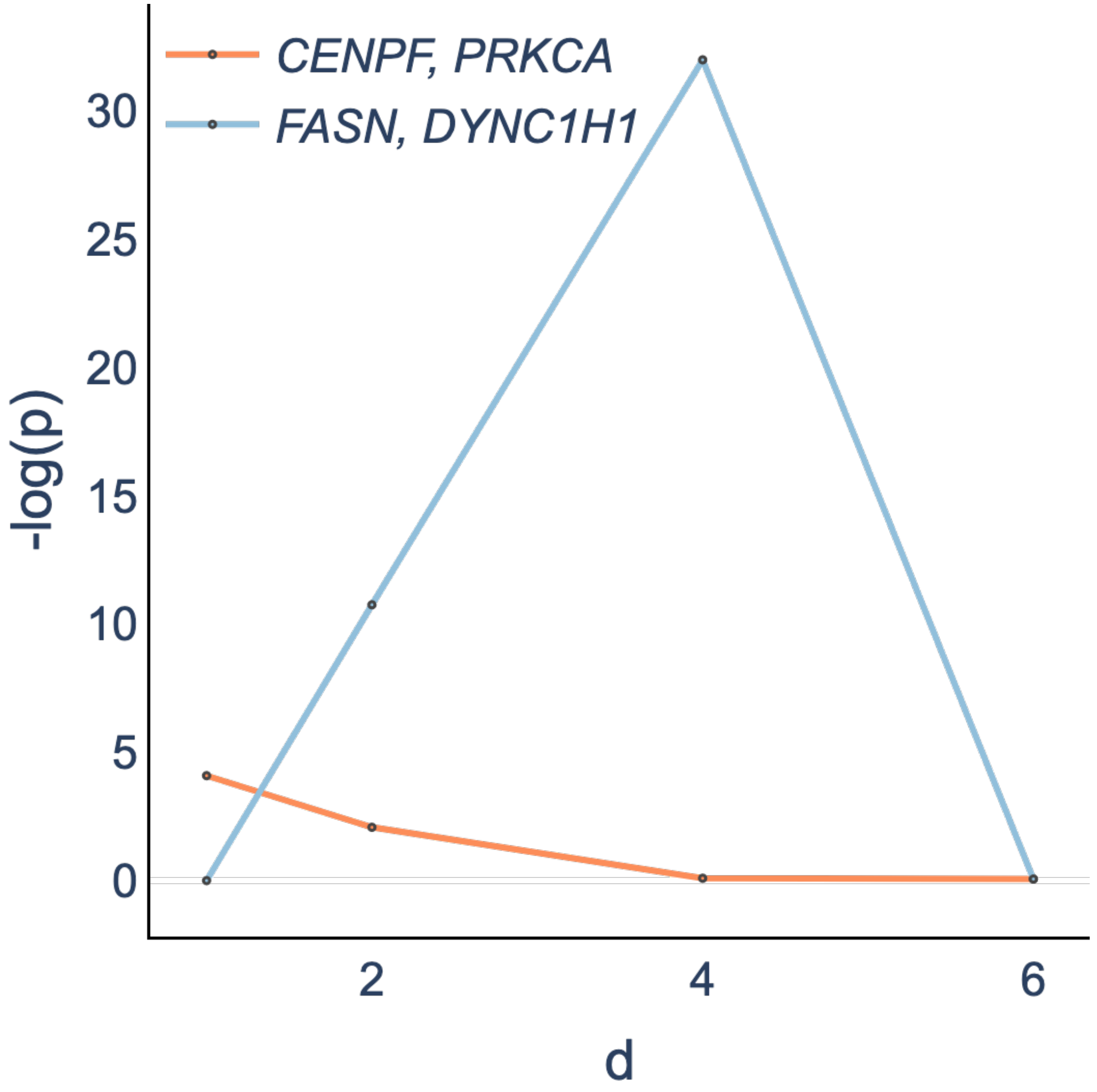

Supplementary Figure 5

Example of scale dependent d-colocalized pairs. Variation of -log(p) from CPB test as *d* varies between 1 and 6 microns for the two gene pairs is shown.

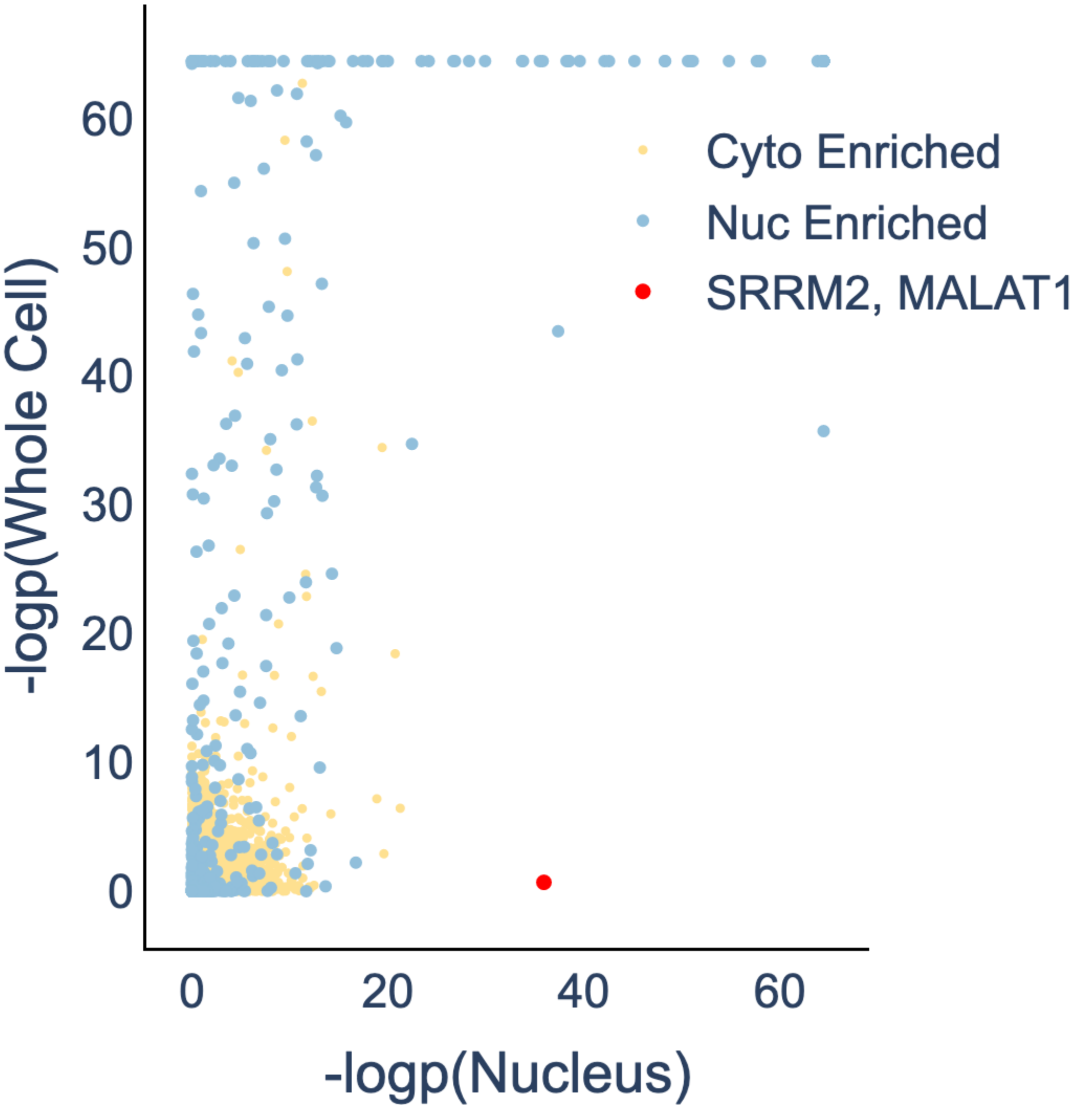

Supplementary Figure 6

Intra-nucleus mode adjusts p-value for nucleus enriched gene pairs. Nucleus enriched pairs is defined as having at least one gene that is among the 3 highest expressed genes in nucleus. Cytosol enriched pairs is defined as having at least one gene that is among the 70 lowest (bottom half) expressed genes in nucleus. Biggest adjustment happens for *SRRM2-MALAT1* which is not d-colocalized at the whole cell level but is colocalized in intra-nucleus mode.

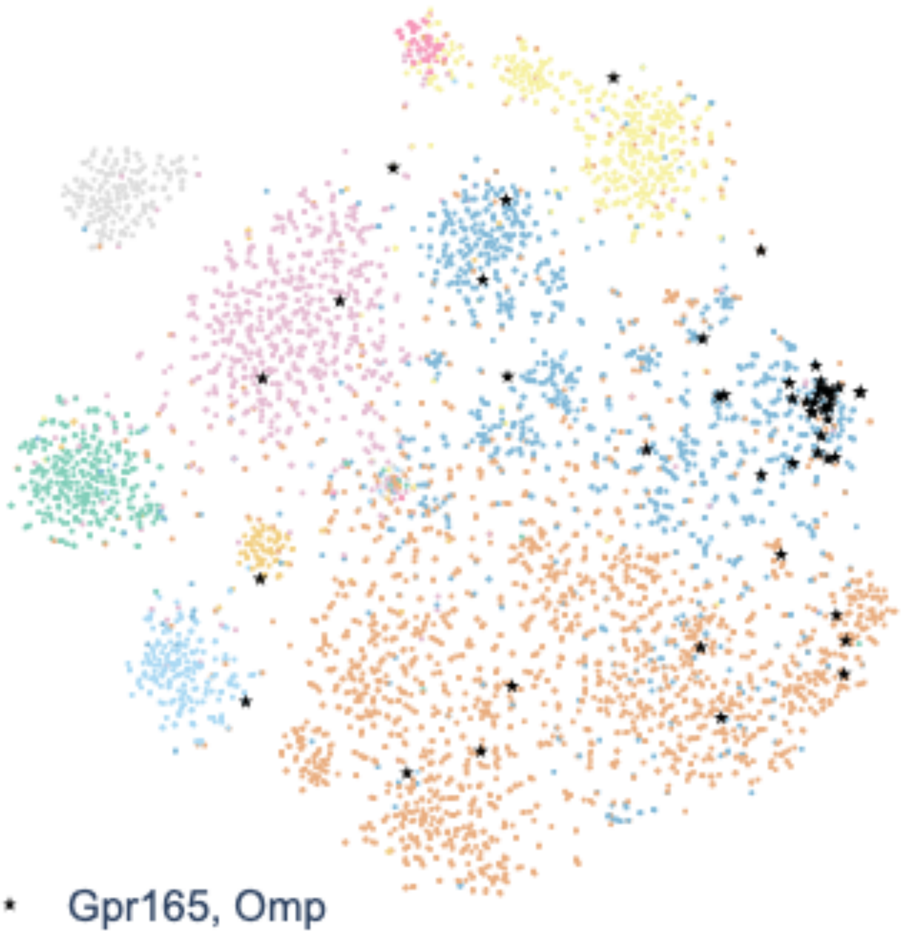

Supplementary Figure 7
Example of a category 2 pair specific to excitatory neurons.

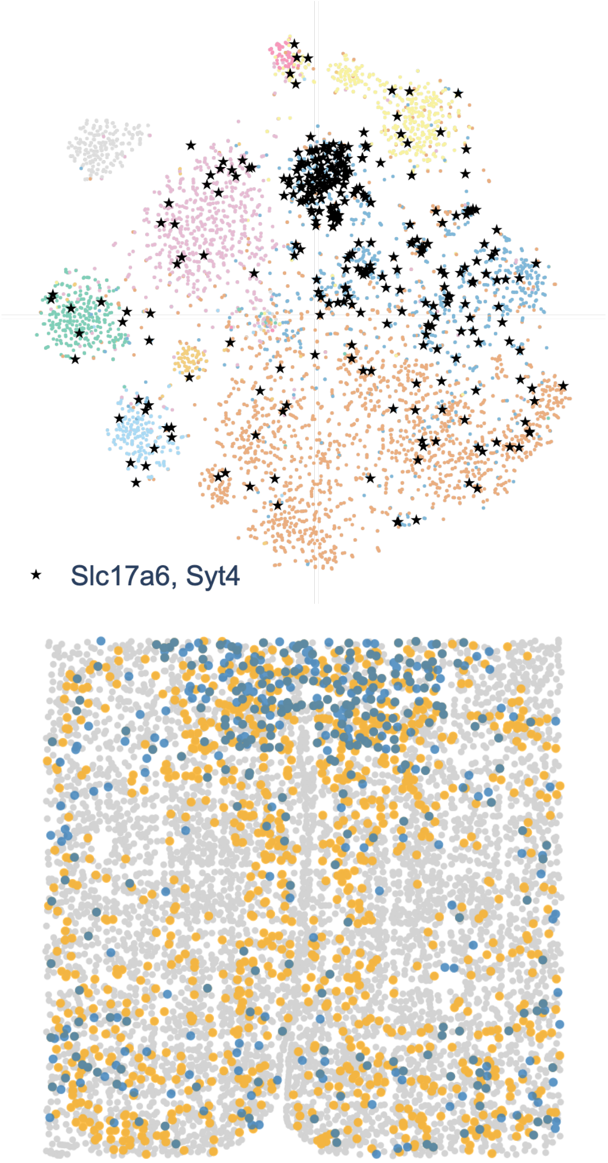

Supplementary Figure 8
Example of a spatially modulated d-colocalized pair *Slc17a6-Syt4* specific to excitatory neurons. Slc17a6 is a vesicular glutamate transporter that is considered a canonical marker of glutamaterigic neurons[1] while Syt4 is a vesicular synaptic protein that regulates Calcium-dependent glutamate release in astrocytes, a gliotransmission pathway that affects synaptic transmission[2].

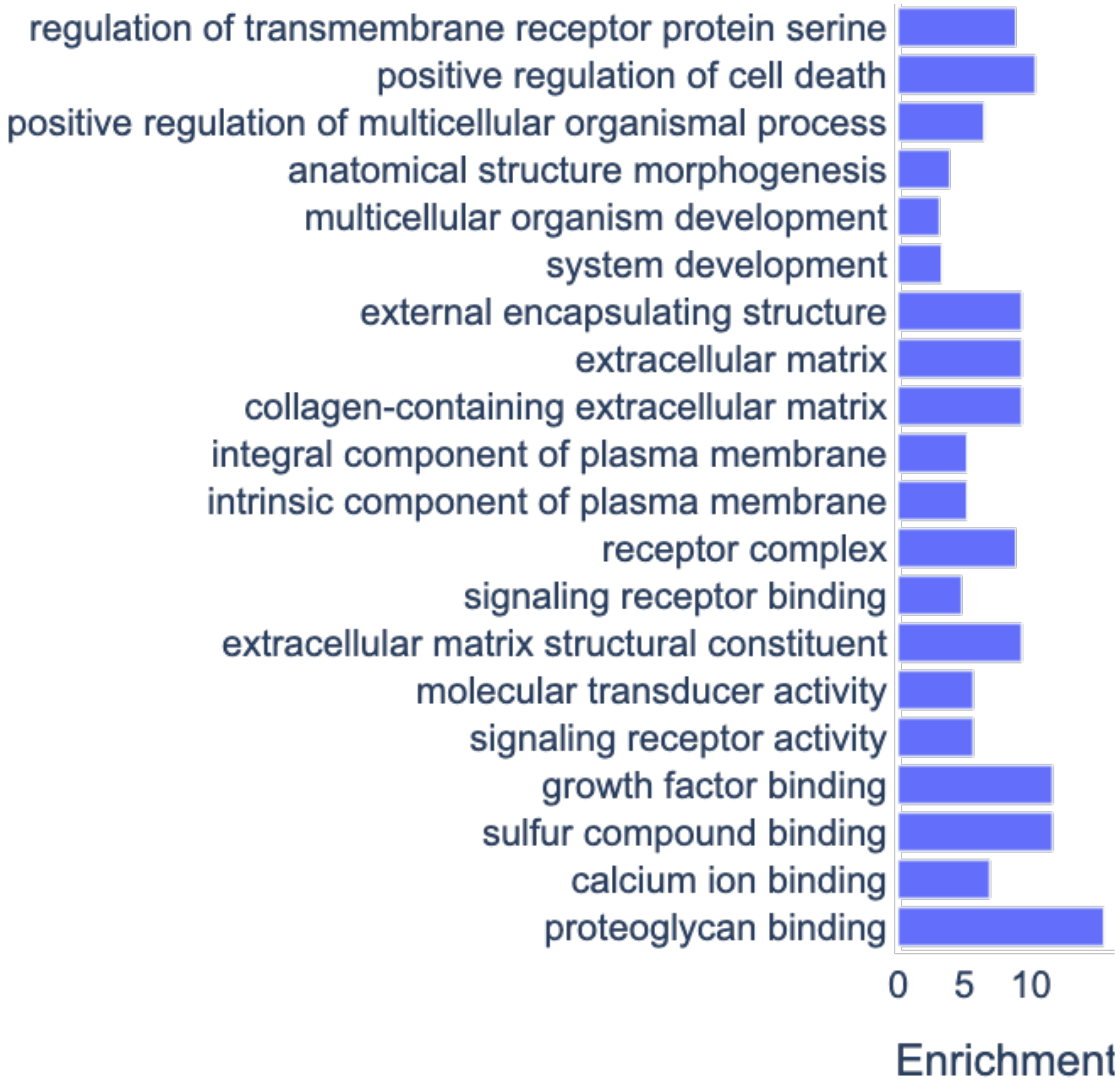

Supplementary Figure 9
GO enrichment analysis of module M2 from Figure 6b.

REFERENCES

1. Vigneault, É. *et al.* Distribution of vesicular glutamate transporters in the human brain. *Frontiers in neuroanatomy* **9**, 23 (2015).
2. Zhang, Q., Fukuda, M., Van Bockstaele, E., Pascual, O. & Haydon, P.G. Synaptotagmin IV regulates glial glutamate release. *Proceedings of the National Academy of Sciences* **101**, 9441-9446 (2004).
